# Tumor fractions deciphered from circulating cell-free DNA methylation for cancer early diagnosis

**DOI:** 10.1038/s41467-022-35320-3

**Published:** 2022-12-13

**Authors:** Xiao Zhou, Zhen Cheng, Mingyu Dong, Qi Liu, Weiyang Yang, Min Liu, Junzhang Tian, Weibin Cheng

**Affiliations:** 1 Department of Automation, Tsinghua University, Beijing, 100084 China; 2 Institute for Healthcare Artificial Intelligence Application, Guangdong Second Provincial General Hospital, Guangzhou, 510317 China

**Keywords:** Machine learning, Diagnostic markers, Cancer

## Abstract

Tumor-derived circulating cell-free DNA (cfDNA) provides critical clues for cancer early diagnosis, yet it often suffers from low sensitivity. Here, we present a cancer early diagnosis approach using tumor fractions deciphered from circulating cfDNA methylation signatures. We show that the estimated fractions of tumor-derived cfDNA from cancer patients increase significantly as cancer progresses in two independent datasets. Employing the predicted tumor fractions, we establish a Bayesian diagnostic model in which training samples are only derived from late-stage patients and healthy individuals. When validated on early-stage patients and healthy individuals, this model exhibits a sensitivity of 86.1% for cancer early detection and an average accuracy of 76.9% for tumor localization at a specificity of 94.7%. By highlighting the potential of tumor fractions on cancer early diagnosis, our approach can be further applied to cancer screening and tumor progression monitoring.

## Abstract (web summary)

‘Circulating cell-free DNA can be used to predict cancer, but it is more challenging to assess in early stage cancer. Here, the authors created a diagnostic model using tumor fractions deciphered from circulating cfDNA methylation signatures, which exhibited an 86% sensitivity in detecting early-stage cancer.

## Introduction

Despite recent advances in cancer treatment, early diagnosis has been conclusively shown to improve the chances of patient survival, and it even offers clinicians the opportunity to cure cancer through the surgical removal of a tumor. However, clinical cancer screening still relies on non-molecular technologies such as gastroscopy, low-dose computerized tomography, and protein biomarkers, while all suffer from low specificity and sensitivity^1–3^. Due to the lack of effective cancer screening modalities, most cancer patients are diagnosed late and thus miss the ideal treatment period. As a liquid biopsy analyte, circulating cell-free DNA (cfDNA) in peripheral blood plasma has emerged as a promising biomarker for cancer early diagnosis due to its non-invasive properties^4–7^. Generally speaking, the cfDNA in the plasma of healthy individuals is derived from hematopoietic cells and normal tissues^8,9^. In contrast, in cancer patients, apart from normal sources, the degraded DNA fragments from tumor cells are released into the bloodstream and constitute a molecularly distinct DNA fragment from total cfDNA^10^. Nevertheless, the fraction of tumor-derived cfDNA relative to the total plasma cfDNA extracted from a cancer patient is not yet abundant enough for routine diagnosis^11^.

To precisely assess tumor-derived cfDNA, copy number variations (CNVs)^12–14^, specific mutations^15–18^ and methylation profiles^19–22^ are broadly exploited as discriminative molecular features of cfDNA. Early tumor fraction prediction approaches^12,13^ based on CNVs relied on costly whole-genome sequencing (WGS) with ~100-fold sequence coverage. The state-of-the-art methods, ichorCNA^14^ and ACE^23^, were developed from low-coverage WGS to quantify tumor fractions in cfDNA. However, both may fail to provide a robust estimate of tumor fractions due to the lack of sufficient aneuploidy and chromosomal instability^24,25^. Although tumor-derived mutations in cfDNA can also be utilized to distinguish potential cancer patients from normal controls, they still remain challenging to detect since mutations vary depending on tumor type, cancer stages (Supplementary Note 1, Supplementary Fig. 1), biological noise, and technical sensitivity^6,26–28^. Moreover, mutation-based diagnostic modalities have great difficulty in localizing the tissue-of-origin (TOO) of tumors, as many driver mutations are shared by multiple malignant tumor types^16^. In contrast, acting as an important epigenetic modification, DNA methylation signatures exhibit significant differences between healthy individuals and those with various diseases, especially malignant tumors^6,29^. As a result, recent studies^30–33^ have analyzed the differentially methylated probes or regions (DMPs/DMRs) that can differentiate healthy individuals from patients with malignant tumors for cancer diagnosis. Since circulating cfDNA has various sources, the methylation level of each CpG site is essentially a mixed signal originating from blood cells and multiple tissues, including tissues that give rise to the cfDNA associated with tumorigenesis^34^. Therefore, it is feasible to estimate the TOO of cfDNA by deconvoluting blended methylation signatures, which may make it possible to predict the location of a primary tumor.

With the advent of machine learning in the field of computational biology, recent studies^31–33,35^ have employed classifiers, such as logistic regression, random forest (RF), and support vector machine (SVM), to construct diagnostic models from cfDNA methylation signatures to detect and localize potential tumors. These data-driven methods, however, are not supported by adequate biological explanations, since an elevated tumor fraction, i.e. the proportion of tumor-derived cfDNA relative to the total cfDNA, essentially shapes the intrinsic characteristics for distinguishing cancer patients from healthy individuals. To estimate the fraction of tumor-derived cfDNA, reference-based deconvolution is the most widely adopted methodology in previous studies^9,34,36^. This approach requires a concatenation of discriminative methylation patterns extracted from each pure tissue to yield a reference database^9^, which is then exploited to deconvolve methylation signatures from circulating cfDNA by solving a nonnegative least square (NNLS) problem. Unfortunately, this approach requires collecting methylation profiles from various cells or tissues to establish a reference database, and it still fails to fully cover the myriad of sources of cfDNA. To address this limitation, the latest method, CelFiE^37^, which was developed using whole-genome bisulfite sequencing data, manages to estimate the proportions of known and unknown cell types from cfDNA methylation profiles. Another tissue-requiring approach is CancerLocator^38^, which constructs a probabilistic distribution for each methylation marker through the convolution of the two Beta distributions fitted from tumor tissue and normal plasma, respectively. Compared with reference-based deconvolution, CancerLocator does not require methylation profiles from normal cells/tissues, which effectively reduces the cost of model construction, but it still needs tumor tissues. Although considerably large datasets of array-based DNA methylation profiles from tumor tissues are available from public resources, such as The Cancer Genome Atlas (TCGA)^39^, plasma data needs to be analyzed from the same methylation sites as the public dataset, which inevitably limits the merits of sequencing-based methylation profiles.

In this work, we present a cancer early diagnosis approach that employs cfDNA methylation profiles to decipher tumor fractions and to localize a potential tumor. Instead of manually concatenating a reference database from tissue data, we propose a semi-reference-free deconvolution (SRFD) algorithm to automatically learn a reference database from cfDNA methylation signatures. With this developed strategy, we observed a significant (*p*-value < 0.005) growth of the estimated tumor fractions with cancer progression in two independent patient plasma datasets including multiple cancer types. Drawing on the advantages of both the tumor fractions and machine learning classifier models, we established a Bayesian diagnostic model, named SRFD-Bayes, to make a final diagnostic decision, and it outperformed the classifier-based diagnostic models on the simulations. Since advanced cancer patients are more common than early-stage patients in clinical practices, we examined patients with late-stage tumors to construct our diagnostic model and validated this model using samples from early-stage patients. Our approach achieved a sensitivity of 86.1% for cancer early detection at a specificity of 94.7%, which outperformed the previous models^33^. Moreover, the average localization accuracy of normal controls and all early tumors reached 76.9%. This represents a significant breakthrough, as the detection sensitivity of machine learning classifiers is often below 72%, while their average localization accuracy on early tumors is less than 55%. In summary, we provide an effective tool that can be applied to monitor tumor progression and has a great potential for large-scale cancer screening.

## Results

### Cancer early diagnosis approach overview

**Fig. 1:**
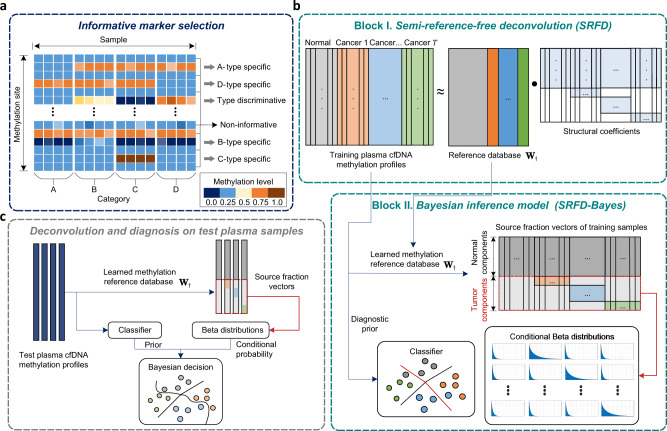
The overview of cancer early diagnosis approach. **a** Informative marker selection. Type-discriminative (TD) and type-specific (TS) markers are screened from methylation sites. **b** Diagnostic model construction. Block I: A methylation reference database, noted by **W**_†_, is first learned from plasma cfDNA methylation profiles by semi-reference-free deconvolution (SRFD), where the structural coefficients are generated from the class label of each training sample. Block II: A Bayesian diagnostic model is constructed for cancer early diagnosis, in which the diagnostic prior is provided by a machine learning classifier established from the training methylation profiles. Meanwhile, the learned reference is employed for the deconvolution of training samples to decipher their source fraction vectors, where each tumor component is then fitted as an independent Beta distribution. **c** Deconvolution and diagnosis on test samples. The test methylation profiles and their corresponding tumor components are separately fed into the learned classifier and the Beta distributions, to obtain a diagnostic prior and a conditional probability, respectively, which contributes to the final Bayesian decision in cancer diagnosis.

Our cancer early diagnosis approach managed to decipher tumor fractions, detect and further localize potential TOO from cfDNA methylation profiles, as depicted in Fig. 1. In the first step, informative markers were selected from a large amount of methylation sites. An informative score (the details of propositions and the corresponding proofs are described in Supplementary Note 2) based on matrix norm was devised to identify type-discriminative (TD) and type-specific (TS) methylation markers (Fig. 1a). Second, instead of manually constructing a reference atlas, we propose to learn a reference database from mixed plasma data using SRFD, in which the class labels imposed structural constraints on the coefficient matrix (block I in Fig. 1b). Third, employing this learned reference database, the training samples were deconvolved into separate source fraction vectors, in which each tumor component was fitted as an independent Beta distribution, while the original methylation profiles were fed into a machine learning classifier (SVM) to construct a pre-diagnostic model, as shown in block II of Fig. 1b.

To further analyze test samples for cancer early diagnosis, the learned methylation reference database was utilized to perform deconvolution and decipher their source fraction vectors (Fig. 1c). Subsequently, the learned SVM classifier provided a diagnostic prior based on the original profiles, meanwhile the fitted Beta distributions yielded a conditional probability from the estimated source fraction vectors. Finally, the prior and the conditional probability were fused to make the Bayesian diagnostic decision for each test sample. The core contributions of our approach mainly consist of the following three aspects: the reference database that was automatically learned by SRFD from plasma cfDNA data instead of tissue data; the Bayesian diagnostic model (SRFD-Bayes) that combined the advantages of both data-driven classifiers and biomedical deconvolution; and the highly transferable training strategy based on advanced tumor samples for early cancer diagnosis.

### Informative methylation marker selection

Since publicly accessible plasma cfDNA methylation data from either healthy individuals or cancer patients is limited, we first collected 656 normal blood DNA methylation profiles from GSE40279^40^, 8 normal plasma pools from GSE12126^9^ and 5 sets of tumor tissue DNA methylation data from TCGA^39^, including Breast Invasive Carcinoma (BRCA), Colon Adenocarcinoma (COAD), Lung Squamous Cell Carcinoma & Lung Adenocarcinoma (LUNG), Liver Hepatocellular Carcinoma (LIHC), and Prostate Adenocarcinoma (PRAD). Secondly, we randomly split these normal blood DNA samples and tumor tissue DNA samples into three sets at a ratio of 4:1:5 to generate simulation datasets for training, validation, and testing, respectively (Supplementary Table 1). The validation dataset was adopted to perform parameter studies while the test dataset was utilized to perform comparative studies with other approaches. Third, to generate simulated cfDNA methylation profiles for cancer patients, we computationally mixed tumor tissue data collected from TCGA and cfDNA data from normal controls at a random ratio, which was consistent with CancerLocator^38^. The numbers of simulated cfDNA data for validation and test were 100 and 400 per category, respectively. More details on the generation of simulation datasets with different levels of CNV events are illustrated in Supplementary Fig. 2, Supplementary Note 3 and Supplementary Tables 2, 3.

**Fig. 2:**
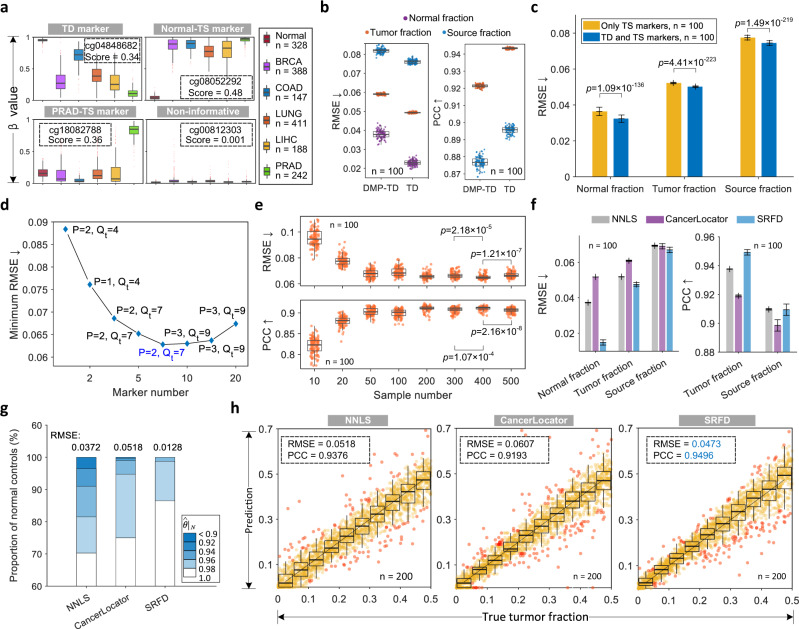
Informative marker selection and deconvolution results on simulations. **a** Representative case of informative markers, including TD and normal/PRAD-TS methylation sites, as well as a non-informative site. *β* value measures the methylation level of a methylation site and Score represents the calculated informative score. The numbers of samples are 328, 388, 147, 411, 188, and 242 for Normal, BRCA, COAD, LUNG, LIHC, and PRAD, respectively. **b** Comparison of the deconvolution performance achieved by NNLS applying differentially methylated probe (DMP-TD) markers and TD markers, respectively. **c** Improvement of root mean square error (RMSE) on the validation dataset after combining TD markers with TS markers. **d** Plot of parameter studies on marker number and pattern number. P and Q_t_ denote the number of normal sources and tumor sources, respectively. **e** Deconvolution results of parameter studies on sample number ranging from 10 to 500 for each category. **f** Comparison of deconvolution performance between different approaches that were evaluated by the predicted normal fractions for healthy individuals, source fractions and tumor fractions for cancer patients, respectively. **g** Comparative performance between different approaches that validated on normal controls. ${\hat{\theta }|}_{N}$ represents the normal fraction calculated by summing the normal components in a predicted fraction vector. The number of normal samples is 400. **h** Scatter and box plots to exhibit the correlations between predicted tumor fractions and their ground truth, in which the black lines and red points denote *y* = *x* and outliers, respectively. The fraction increment and sample number of each boxplot is 0.05 and 200. Two-sided Welch’s *t* test is used to assess the statistical significance of performance difference in **c** and **e**. *n* = 100/200 independent repeats across all experiments. Error bars (in mean and standard deviation) were obtained by statistically repeating experiments 100 times in **c** and **f**. The boxes in **a**, **b**, **e**, and **h** are bounded by the first and third quartile with a horizontal line at the median and whiskers extend to the maximum and minimum values. Source data are provided as a Source Data file.

To select TD and TS methylation markers for each category, we developed an informative score based on matrix norm (see Methods and Supplementary Note 2) and applied it to the simulation datasets. Figure 2a and Supplementary Fig. 3 illustrate the Top-1 TD, the Top-1 TS methylation markers and one non-informative site ranked by the informative score. Most categories were differentially distributed on the TD marker cg0484862, while a clear gap lay between the specific type (normal/PRAD) and all other categories on the TS markers (cg08052292/cg18082788). As for the non-informative site, all classes, as expected, shared no distribution differences. It could be further observed that the informative score of the Top-1 TD marker was lower than that of the normal/PRAD-TS marker. This was largely because the *β* value of methylation signatures was normalized in [0, 1] and presented a bimodal distribution. As a result, increasing the number of tumor classes would lead to overlapped distributions, which decreased the discriminability of a specific methylation site.

To demonstrate the advantages of TD markers selected based on matrix norm (noted as TD), we compared them with DMP-TD markers, which were identified by the intersection of DMPs in every two categories, and ran NNLS to evaluate their deconvolution performance. Figure 2b shows the comparative results employing the Top-500 TD and Top-500 DMP-TD markers, in which normal and tumor fractions were calculated by summing the normal and abnormal components of the fraction vector, respectively. The source fractions were computed by summing the corresponding components for each tumor type. We observed that our TD markers achieved a lower root mean square error (RMSE) for both normal fractions of normal controls and tumor/source fractions of cancer patients, which furthermore raised the Pearson Correlation Coefficients (PCC) of the latter by 4%, suggesting that our TD markers outperformed DMP-TD markers for deconvolution tasks. Moreover, TD markers only required to traverse each marker candidate once while the computational cost for DMP-TD markers increased with category number. To further evaluate the effectiveness of TD markers, we repeated NNLS 100 times, each with a different reference, to quantify the deconvolution performance with and without TD methylation sites. It can be concluded from Fig. 2c that after combining TD markers, the average RMSE declined on all predicted fractions, especially for normal fractions whose RMSE decreased by 8%, suggesting that TD markers contributed to a more precise deconvolution of mixed methylation signatures.

### Deconvolution performance on a simulation dataset

Instead of manually constructing a methylation reference atlas from massive cell types and tissues, we proposed to learn a reference database by performing SRFD on cfDNA methylation signatures. The details of SRFD are described in the Methods section. There were three critical parameters during the training process of SRFD, including marker number, methylation pattern number and plasma sample number, which were configured by an elaborate parameter study. We first explored the influence of the marker number and methylation pattern number on deconvolution, meanwhile fixing the plasma sample number of each category to 1000 to simulate a scenario with sufficient samples. Supplementary Fig. 4 and Fig. 2d separately display all RMSE and minimum RMSE values among multiple parameter groups with different marker and pattern numbers. Accordingly, the deconvolution performance first improved and then descended as the number of markers increased while achieving the lowest RMSE when the Top-50 markers were selected for each category. Meanwhile, the best performance on the validation dataset was achieved when the numbers of normal and tumor patterns were set to 7 and 2, respectively. As a result, we configured them as 7 and 2 throughout all the experiments in this study.

Subsequently, we explored the influence of training sample number and plotted the trend of deconvolution performance with an increasing number from 10 to 500 for each category, as shown in Fig. 2e. It can be concluded that the RMSE significantly decreased as the number of training plasma samples grows, and then gradually levelled off when more than 200 samples were adopted. To ensure a robust performance, we chose 400 plasma samples for each category in the following deconvolution experiments on simulation datasets. The advantages of our approach mainly consisted of two aspects. First, only plasma data was required to yield the reference database, which greatly decreased the cost of tissue collection. Second, more than one methylation pattern was finally obtained for an individual cancer type, which might reveal the heterogeneity of malignant tumors. The configuration of other parameters in SRFD is described in Supplementary Note 4.

We next compared SRFD with other up-to-date approaches, including reference-based NNLS^9^ and CancerLocator^38^ (see the implementation details in Supplementary Note 5 and Supplementary Fig. 5). Because the requiring training samples are different between reference-based approaches (cfDNA methylation from healthy controls and tissue DNA methylation from cancer patients) and SRFD (only cfDNA from normal individuals and cancer patients), the training tissue data used for reference-based methods was also utilized to generate the corresponding simulated plasma data to ensure a fair comparison (Supplementary Fig. 2).

The evaluation of predicted normal fractions for healthy individuals and tumor/source fractions for cancer patients among different approaches is shown in Fig. 2f (30% CNV) and Supplementary Fig. 6 (10% and 50% CNVs). We found that the RMSE of predicted tumor fractions was much lower than that of the source fraction over all approaches, suggesting that the tumor fraction was more appropriate to predict the true fraction. SRFD outperformed CancerLocator and NNLS on both normal and tumor fractions by a large margin, which highlighted that the methylation reference database learned from mixed plasma data generated more precise estimate of tumor fractions. More specifically, a comparative study on normal controls is shown in Fig. 2g, in which 87% of healthy controls were estimated with a normal fraction greater than 0.98 by our approach, while less than 75% was covered by CancerLocator or NNLS. In particular, our predicted normal fractions of healthy individuals were barely lower than 0.94, in contrast, NNLS provided more than 3% of healthy controls with <0.92 estimated source fractions. The overall RMSE of our approach on normal controls was 0.0128, which was remarkably less than one-third that of NNLS and one-fourth that of CancerLocator. This demonstrated that when employing the same methylation markers, our learned reference database contributed to a better deconvolution performance on normal controls compared with a manually constructed reference database.

With respect to the simulated cfDNA data for cancer patients, predicted tumor fractions should be equal to the corresponding pre-set tumor fractions under different levels of CNV events, as shown in Fig. 2h (30% CNV) and Supplementary Fig. 7 (10% and 50% CNVs). It can be noted from the scatter and box plots that the tumor fractions deciphered by NNLS and CancerLocator exhibited a large number of outliers. Compared with the abovementioned approaches, the RMSE between our prediction and the true fractions under 30% CNV events had dropped by 8.7% and 22.1%, respectively. Moreover, our approach also achieved the highest PCC across all CNV events.

### Diagnostic performance on a simulation dataset

To consolidate the biological explanation of data-driven SVM classifiers, the tumor fraction deciphered from methylation profiles was introduced to establish a Bayesian diagnostic model, named SRFD-Bayes, which is detailed in the Methods section. Since, practically speaking, the number of plasma samples from cancer patients fulfilling inclusion criteria was very limited, we first designed an experiment, named Diagnosis A, in which 40 cases from the simulated samples of each tumor type were randomly selected to imitate a limited number of cancer patients. Correspondingly, a total of 400 samples were utilized to construct a diagnostic model that was evaluated on test datasets including 2400 samples, as summarized in Supplementary Table 3. We repeated every experimental group 100 times, each with a random training dataset, to determine the average performance as well as the robustness.

**Fig. 3:**
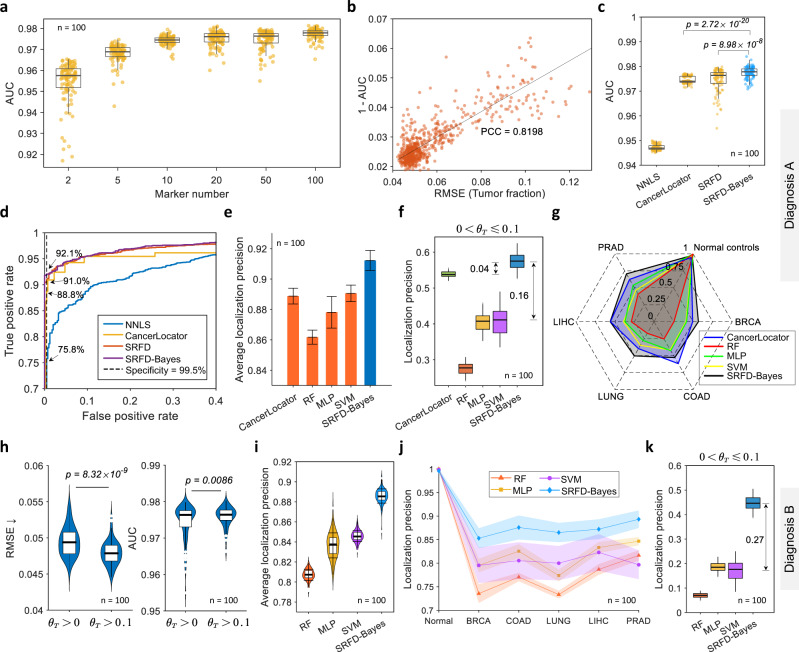
Diagnostic results on the simulation dataset. **a** Area under curve (AUC) of cancer detection, achieved by tumor fraction, among different numbers of markers validated on the simulation dataset. **b** Correlation between RMSE of estimated tumor fraction and one minus its corresponding AUC. **c** The performance comparison of cancer detection between different approaches, while the orange groups are achieved by the cut-off of tumor fraction and the blue one denotes the results of our Bayesian diagnostic model. **d** Receiver operating characteristic curves (ROCs) of the median results shown in **c**. **e** Comparison of average localization precision between Bayesian diagnostic model and other approaches, including CancerLocator, random forest (RF), multi-layer perception (MLP) and support vector machine (SVM). Error bars (in mean and standard deviation) were obtained by statistically repeating experiments 100 times. **f** Comparison of localization performance on cancer samples with tumor fraction less than 0.1 (0 < *θ*_*T*_ ≤ 0.1). **g** Radar plot of the localization precision on each category achieved by different approaches. **h** Accuracy comparison of predicted tumor fraction, evaluated by RMSE (left) and AUC (right), when using tumor samples with all tumor fractions (*θ*_*T*_ > 0) and with tumor fraction higher than 0.1 (*θ*_*T*_ > 0.1) as training dataset, respectively. **i** The average localization precision and **j** its distribution on different categories achieved by different approaches when using cancer samples with *θ*_*T*_ > 0.1 as training dataset. Error bands (in mean and standard deviation) were obtained by statistically repeating experiments 100 times. **k** The comparison of localization performance tested on cancer samples with 0 < *θ*_*T*_ ≤ 0.1. The number of test samples for each category is 400 in both Diagnosis A and B, while the number of test samples with 0 < *θ*_*T*_ ≤ 0.1 is 80 for each type of tumor. Two-sided Welch’s *t* test is used to assess the statistical significance of performance difference in **c** and **h**. *n* = 100 independent repeats. The boxes are bounded by the first and third quartile with a horizontal line at the median and whiskers extend to the maximum and minimum values. Source data are provided as a Source Data file.

Next, to evaluate the capability of the estimated tumor fraction on cancer detection, we first utilized the tumor fraction to directly distinguish cancer patients from healthy individuals and adopted the area under curve (AUC) to quantify the detection performance. Figure 3a illustrates that the AUC increased as the number of markers grew, which, to a large degree, matched the decreasing trend of a minimum RMSE with an increasing marker number in Fig. 2d. Moreover, a positive correlation (PCC = 0.8198) occurred between the deconvolution metric RMSE and the detection metric 1 - AUC, as shown in Fig. 3b, suggesting that the AUC can also be used to quantify the accuracy of predicted tumor fractions, especially for diagnosis using patient cfDNA since their tumor fraction may be unquantifiable. Figure 3c shows the comparative performance on cancer detection between different approaches, where the orange and the blue dots represent the experimental results achieved by estimated tumor fractions and SRFD-Bayes model, respectively. It can be concluded that the tumor fraction deciphered by CancerLocator and SRFD achieved comparable median performances, which outperformed NNLS by a large margin, while SRFD suffered from a larger standard deviation. This was because SRFD directly handled plasma cfDNA to construct a reference matrix, which could be undermined by cancer samples with considerably low tumor fractions due to their similarity with normal controls. Nevertheless, after performing Bayesian inference, SRFD-Bayes significantly improved the robustness and further achieved a higher median AUC that was close to 0.98. The median ROC curves of all approaches are shown in Fig. 3d, in which a desired specificity of 99.5% was selected to compare the sensitivity of cancer detection. It is clear that SRFD-Bayes achieved the highest sensitivity of 92.1%, which outperformed CancerLocator and NNLS by 3.3% and 16.3%, respectively.

Consequently, we compared SRFD-Bayes with CancerLocator and other popular machine learning classifiers, including RF, multi-layer perception (MLP) and SVM methodologies to evaluate the cancer localization performance. The machine learning classifiers shared the identical training samples with SRFD-Bayes. The localization accuracy, computed by the mean of the classification accuracy for each category, was adopted to quantify the final performance. A comparison on all test samples achieved by different methods is shown in Fig. 3e. It was clear that SVM and CancerLocator outperformed the other two classifiers and achieved a comparable diagnostic accuracy of approximately 0.89. However, after integrating Bayesian inference based on the estimated tumor fractions, SRFD-Bayes achieved the best localization precision, which was above 0.91.

Furthermore, to demonstrate the localization performance of our approach for recognizing cancer patients with different levels of tumor fractions, which may be related to tumor progression, we divided test cancer samples into five subsets with increasing tumor fractions. Figure 3f and Supplementary Fig. 8 individually show the performance on different tumor fractions with 0.1 intervals. Interestingly, all the approaches achieved a comparable localization precision of more than 0.92 on the cancer samples with a tumor fraction higher than 0.1 (*θ*_*T*_ > 0.1). However, a significant difference occurred in the group with a tumor fraction less than 0.1 (*θ*_*T*_ ≤ 0.1), as shown in Fig. 3f, where CancerLocator and our diagnostic model outperformed other data-driven classifiers by at least 0.13 precision. There were two reasons behind this improvement. First, cancer samples with *θ*_*T*_ ≤ 0.1 shared very similar features with normal controls and the number of normal controls was much larger, therefore the data-driven classifiers were more inclined to misdiagnose these samples. More importantly, both CancerLocator and SRFD-Bayes managed to excavate hidden information, i.e. tumor fraction, to magnify the difference between normal controls and cancer samples. Consequently, they were more sensitive to cancer samples with low tumor fractions. Meanwhile, SRFD-Bayes still achieved more promising performance than CancerLocator for the cancer samples with *θ*_*T*_ > 0.1, which was beneficial from classifiers. In summary, SRFD-Bayes combined both data-driven methods and biomedical information to improve diagnostic performance. Consistently, the radar plot in Fig. 3g shows the average localization accuracy of different approaches on normal controls and the cancer patients with *θ*_*T*_ ≤ 0.1, in which all classifiers, including RF, MLP and SVM, almost precisely diagnosed every normal control, while their localization accuracy on tumor samples remained considerably limited. After introducing tumor fractions, the localization accuracy of SRFD-Bayes on normal controls barely decreased, while that on all other tumors increased. In particular, nearly 75% of PRAD patients with *θ*_*T*_ ≤ 0.1 were precisely localized using SRFD-Bayes.

Considering the fact that plasma samples from advanced cancer patients were more accessible than those from early-stage patients, we designed another experiment, named Diagnosis B, in which only 32 cases with *θ*_*T*_ > 0.1 of each cancer type were selected during the training process to simulate this practical limitation. Figure 3h exhibits the accuracy of predicted tumor fractions between Diagnosis A and B. Both the RMSE and the AUC did not deteriorate but rather improved when using cancer samples with *θ*_*T*_ > 0.1 for training. In addition, the localization performance achieved by different methods is shown in Fig. 3j. We observed that SRFD-Bayes still outperformed classifiers by a large margin on the average and had better-categorized localization precision. When focusing on test patients with *θ*_*T*_ ≤ 0.1, the localization precision of SRFD-Bayes significantly outperformed other popular classifiers by at least 0.2, as shown in Fig. 3k, enlarging the performance gap in Fig. 3f. This was due to the generalization ability of machine learning classifiers being highly reliant on the distribution of the training dataset. Therefore, they struggled to recognize early-stage cancer patients that they had not met during training. In contrast, SRFD-Bayes combined biomedical explanations with data-driven classifiers to make a final diagnostic decision. As a result, Diagnosis B inspired a training strategy to diagnose early-stage cancer patients while training the diagnostic model on advanced tumor samples for following experiments on patient cfDNA data.

### Deconvolution and diagnostic results on patient cfDNA

**Fig. 4:**
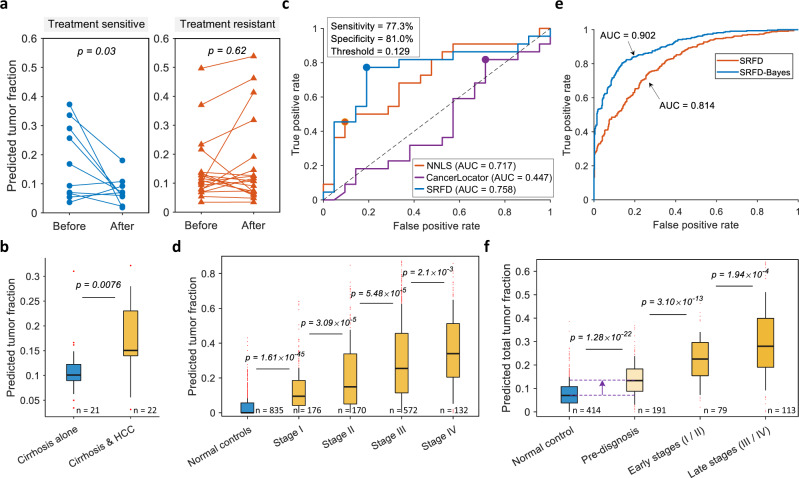
Deconvolution results on patient plasma cfDNA. **a** Alteration of predicted tumor fractions before and after abiraterone acetate (AA) treatment on the patients with prostate cancer. The left graph suggests that confirmed treatment-sensitive patients show a significant decline on tumor fraction after the AA treatment, while no such phenomenon appears in the treatment-resistant patients (Right). Two-sided Welch’s *t* test for paired samples is used to assess the statistical significance. **b** Comparison of predicted tumor fractions between cirrhosis patients and the patients with cirrhosis as well as hepatic carcinoma (HCC). **c** Comparison of ROC curves calculated by exploiting predicted tumor fractions to classify cirrhosis patients and the patients with cirrhosis as well as HCC. **d** Significantly differential distribution (*p*-value < 0.003) of predicted tumor fraction among normal controls and HCC patients with different cancer stages, in which the median of predicted tumor fractions increases as cancer progresses. **e** Comparison of ROC curves calculated by SRFD and SRFD-Bayes when distinguishing HCC patients from normal controls. **f** Significantly differential distribution (*p*-value < 0.001) of predicted tumor fractions among normal controls, pre-diagnosis (asymptomatic participants who were later diagnosed with cancer in the following one to four years) and confirmed patients with early/late-stage cancers. The number of samples from different categories is shown below each box. The boxes are bounded by the first and third quartile with a horizontal line at the median and whiskers extend to the maximum and minimum values. Two-sided Welch’s *t* test is used to assess the statistical significance of predicted tumor fractions among different stages in **b**, **d** and **f**. Source data are provided as a Source Data file.

To validate the reference database learned from the simulation dataset for deconvolution, we directly applied it to deconvolve patient plasma cfDNA methylation profiles. The patient cfDNA datasets adopted in this study are summarized in Supplementary Table 4. First, we exploited the learned reference database, without any modification, to perform deconvolution on 10 treatment-sensitive and 19 treatment-resistant PRAD patients from GSE108462^41^. Similarly, we also found that the estimated tumor fractions of most treatment-sensitive patients dropped significantly (*p*-value = 0.03) after treatment, while those of treatment-resistant patients did not show a significant difference (*p*-value = 0.62) and a few patients even exhibited increasing tumor fractions after treatment, shown in Fig. 4a. Our learned reference database was also employed to deconvolve a cfDNA methylation dataset from 21 cirrhosis patients and 22 patients with both cirrhosis and hepatic carcinoma (HCC) from GSE129374^42^. The difference in the predicted tumor fractions between the patients with only cirrhosis and the patients with both cirrhosis and HCC was statistically significant (*p*-value = 0.0076), as shown in Fig. 4b. This suggested that the plasma of the latter contained more liver/tumor-derived cfDNA than the cirrhosis patients, which provides a meaningful biological explanation to distinguish HCC patients from cirrhosis patients via the use of cfDNA methylation profiles. The comparison of ROC curves calculated by exploiting predicted tumor fractions to classify patients with HCC as well as cirrhosis and cirrhosis patients is shown in Fig. 4c, in which the diagnostic performances of NNLS and CancerLocator were also evaluated using their tumor fractions. Although the tumor fraction distributions of cirrhosis and HCC patients overlapped, the best cut-off value (0.129) of SRFD could still reach a sensitivity of 77.3% at a specificity of 81.0%, which outperformed NNLS and CancerLocator by a large margin. To demonstrate the influence of marker and sample numbers on patient cfDNA, we also summarized the comparative AUC values in Supplementary Fig. 9, which exhibited a consistency with Fig. 2d, e.

Subsequently, we utilized plasma cfDNA signatures from a large cohort^31^, including 835 normal controls and 1050 HCC patients at known cancer stages, to decipher their tumor fractions. Since each plasma sample only contained 10 methylation sites, which did not overlap with the markers we selected from simulation datasets, we randomly selected a half of normal controls and the patients with late cancer stages (III/IV) to learn another methylation reference database. Afterwards, the reference database was employed to run deconvolution on these methylation signatures and the experimental results are displayed in Fig. 4d. It can be concluded that the estimated tumor fraction of HCC patients was conspicuously higher than that of normal controls, and more importantly, the tumor fractions were observed to be significantly (*p*-value < 0.003) correlated with cancer stages. The median of the predicted tumor fractions gradually increased as the disease deteriorated, indicating that the progression of cancer would coerce the tumor/normal tissue to release more DNA into the peripheral blood. Similarly, we directly employed the tumor fractions predicted by SRFD to distinguish HCC patients from healthy individuals, and then further trained SRFD-Bayes model on normal controls and patients with late stages (III/IV) to validate the performance on patients with early stages (I/II). The corresponding ROC curves are presented in Fig. 4e. It can be observed that the classification performance of predicted tumor fractions was very limited, while after applying our diagnostic model, the AUC reached 90.16% (i.e. 8.6% improvement). Based on the previous parameter studies in simulations, the marker number was a critical aspect affecting the deconvolution and detection performance. Therefore, we believe that an increasing number of markers in this dataset could improve the performance of distinguishing early-stage HCC patients from normal controls.

To evaluate our cancer early diagnostic approach on different cancer patients, we collected plasma cfDNA signatures^33^ containing 414 normal controls, 223 cancer patients (colorectal 7, liver 23, esophageal 68, lung 56, and stomach 69) with stage labels and 191 pre-diagnosis patients (asymptomatic participants who were later diagnosed with cancer in the following one to four years). In practice, early-stage tumors usually lead to very mild symptoms that are easily ignored. Accordingly, it is difficult to recruit enough early-stage cancer patients unless a large-scale cohort study is carried out. Unfortunately, at present, there is still no reliable clinical cancer screening modality with high sensitivity. In this study, we adopted the patients with late-stage tumors (III/IV) and a random half of normal controls to construct SRFD-Bayes diagnostic model. The tumor fractions for samples in different disease situations were first deciphered using SRFD, as shown in Fig. 4f, where an apparent difference was observed between normal controls and cancer patients. The mean tumor fraction of early samples was significantly (*p*-value < 0.001) lower than that of patients with late-stage tumors. Moreover, the predicted tumor fraction of pre-diagnosis patients was exactly located between that of normal controls and that of confirmed early-stage patients. It should be noted that asymptomatic participants were not diagnosed with any cancers when they were recruited. Their tumor fractions were consequently supposed to be significantly lower than that of confirmed cancer patients. Due to their later diagnosis with malignant tumors in the following one to four years, there might be a slight difference between their plasma cfDNA signatures and those of normal controls. This difference was presented in the predicted tumor fractions, as depicted in Fig. 4. Overall, these discoveries demonstrated that the predicted tumor fraction could potentially reveal cancer progression in a non-invasive manner.

**Fig. 5:**
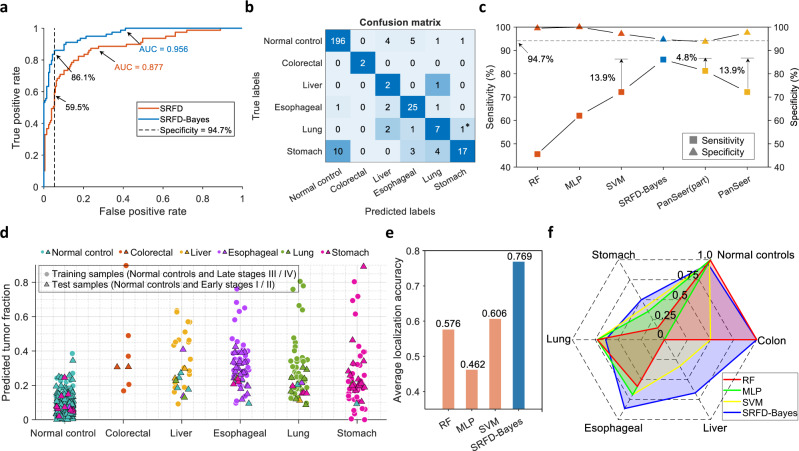
Diagnostic results on patient plasma cfDNA methylation profiles. **a** Comparison of ROC curves calculated by SRFD and SRFD-Bayes when distinguishing cancer patients from normal controls. **b** Diagnostic results, illustrated by confusion matrix, on normal controls and early-stage cancer patients. Only late-stage cancer patients and a random half of normal controls are utilized as training samples for model establishment. **c** Comparison of detection sensitivity and specificity on early-stage cancer patients between SRFD-Bayes approach and other machine learning classifiers. PanSeer (part) denotes the cancer early diagnostic results from the original study^33^. PanSeer represents the reproduced diagnostic results under our data-split strategy. **d** Visualization of predicted tumor fractions of training samples (207 normal controls and 113 late-stage patients marked by colorful circulars) and test samples (207 normal controls and 79 early-stage cancer patients marked by colorful triangles). **e** Comparison of average localization accuracy on normal controls and early-stage cancer patients between different approaches. **f** Localization accuracy of each category achieved by different approaches. * represent a lung cancer patient with unknown stage. Source data are provided as a Source Data file.

Secondly, we exploited the estimated tumor fractions of our training samples, including all patients in late stages and half of normal controls, to establish a Bayesian diagnostic model for cancer diagnosis, and then validated this diagnostic model on all early-stage patients and the remaining half of normal controls. Since the number of training samples in each category was severely imbalanced (only 5 cases for colorectal cancer, while 45 and 207 cases for lung cancer and normal controls, respectively), we utilized a synthetic minority oversampling algorithm (Methods) based on the source fraction vectors. With this strategy, the categories that contained less than 40 samples were oversampled to 40 cases and thus all tumor types achieved a data balance, while the number of total cancer patients was approximately equal to that of normal controls. The comparison of ROC curves calculated by SRFD and SRFD-Bayes on cancer detection is shown in Fig. 5a. Since the marker number in this dataset was much larger than that in the former HCC dataset^31^, the AUC of tumor fractions predicted by SRFD achieved 0.877, which was 6.3% higher than that in Fig. 4e. Additionally, SRFD-Bayes achieved much better performance than the predicted tumor fraction, where the sensitivity of our diagnostic model reached 86.1% at a specificity of 94.7%. In contrast, the sensitivity of SRFD was less than 60% at the same cut-off threshold. The influence of different sample numbers for the training period of SRFD and SRFD-Bayes was also evaluated in both patient cfDNA datasets^31,33^ and presented in Supplementary Fig. 10.

The diagnostic results of early-stage patients and normal controls are summarized in a confusion matrix, as depicted in Fig. 5b. It can be stated that only 11 normal controls were misdiagnosed and 11 early-stage patients were mis-detected, indicating an ideal specificity of 94.7% and a sensitivity of 86.1% for cancer early detection. However, the original study PanSeer (part)^33^, in which a random half of the data with all stages was utilized for training, achieved a specificity of 93.7% and a sensitivity of 81.3% (Fig. 5c) for cancer early diagnosis (calculated from its supplementary materials). To achieve a fairer comparison, we reran PanSeer, whose key algorithm is logistic regression for cancer detection, with our data-split strategy that late-stage patients for training and early-stage patients for validation. It can be observed from Fig. 5c that PanSeer suffered from false negatives and thus detected only 72.2% of early-stage cancer patients. The pre-diagnosis participants were additionally tested by SRFD-Bayes, as it was discovered that only 73 asymptomatic cases were diagnosed with cancer (Supplementary Fig. 11). Moreover, compared with the diagnostic results predicted by the machine learning classifiers (Supplementary Fig. 12b), our approach still achieved 0.405, 0.241 and 0.139 higher sensitivity than RF, MLP and SVM, respectively (Fig. 5c). The predicted tumor fraction of every plasma sample is shown in Fig. 5d, where the early-stage patients were mainly located at the low-fraction area of the distribution range for each tumor type. Figure 5e displays the comparison of average localization accuracy between our approach and other classifiers, while SRFD-Bayes model outperformed other approaches by a large margin and the average localization accuracy reached 76.9%. Fig. 5f and Supplementary Fig. 12 show more specific comparison of localization accuracy for each category. We observed that SRFD-Bayes achieved the best localization accuracy for most tumor types. Compared with classifiers that were inclined to misdiagnose cancer patients, especially for esophageal and stomach cancers, SRFD-Bayes had slightly degraded accuracy on lung cancer patients but significantly reduced false negatives for other cancers. This decrease was caused by the fact that tumor fraction vectors of test lung samples improperly distributed on other tumor components, suggesting that different tumor samples might share similar methylation features. The above experimental results demonstrated that SRFD-Bayes could be applied to establish a reliable cancer early diagnostic model with employing cancer samples from only late-stage patients, which can shorten the period for clinical recruitment.

Finally, to show the advantages of our model construction strategy, we designed a control experiment in which a random half of all categories were randomly selected as training samples for ten repeats. The diagnostic performance is shown in Supplementary Fig. 13a, where it is clear that our synthetic oversampling strategy could still increase the diagnostic accuracy of all approaches. In addition, compared with our strategy, in which only normal controls and late-stage patients were adopted for model training, the performance of MLP, and SVM in the control experiment achieved a conspicuous enhancement (Supplementary Fig. 13b). The reasons for this phenomenon might be that, with respect to data-driven classifiers, randomly dividing the dataset can provide a more consistent data distribution between training and test samples. As a result, early cases in the training dataset could promote the diagnostic accuracy on early-stage patients. Meanwhile, the diagnostic accuracy of SRFD-Bayes dropped from 0.78 to approximately 0.67, but still outperformed individual classifiers.

## Discussion

Reference-based deconvolution approaches require the methylation signatures of pure tissues to manually construct a reference atlas, which can be confounded by factors such as age or genotype^43^. On the contrary, reference-free algorithms^44,45^ directly address mixed data, but they cannot estimate the source fractions of individual samples^43^. In this study, we propose a semi-reference-free deconvolution approach based on nonnegative matrix factorization^46^ to automatically learn a methylation reference database, which only requires methylation signatures from mixed plasma cfDNA. Without directly handling tissue data, our method is not restricted by the assumption that the distribution of methylation signals detected from tumor-derived cfDNA is identical to that from the corresponding tumor tissue, which is the basis of reference-based approaches. Consequently, our approach can establish a reference with a higher fidelity. In contrast to reference-free approaches, we introduce class labels of training samples to shape the structural supervision for the coefficient matrix. As a result, the methylation patterns for each category can be separately arranged in a pattern matrix, which acts as the final methylation reference database for the source fraction prediction of cfDNA. Considering that it was unnecessary to explore specific normal sources of cfDNA when predicting tumor fractions, our approach could effortlessly construct multiple normal sources by adjusting the number of methylation patterns for normal controls. Moreover, the learned multiple patterns for individual tumor sources, to a certain extent, could reflect tumor heterogeneity and a further analysis on the learned tumor patterns might provide valuable clues for the causes and consequences.

In this study, deconvolution results of cfDNA methylation signatures extracted from cancer patients demonstrated that the estimated tumor fraction of HCC patients was significantly higher than that of cirrhosis patients, which could contribute to distinguishing non-cancer diseases from malignant tumors. In addition to the difference of tumor fractions observed between normal controls and patients with diseases^37^ /tumors^9^, we also found a significant growth of tumor fractions as the cancer stage progressed in two independent cfDNA methylation datasets, which may inspire an alternative approach for monitoring tumor progression. It is noteworthy that the cfDNA methylation data adopted in this study came from different platforms, including array-based and bisulfite sequencing methods. Essentially, our approach only required the (average) methylation level of each site (region) to perform deconvolution and subsequent analysis, thus it was protected from differences in the data acquisition platforms. Since genomic CNVs can lead to a change in DNA methylation^34^, a potential improvement to our approach would be simultaneously deciphering both the tumor fractions and CNVs from circulating cfDNA methylation signatures. Combining these two informative features might further improve deconvolution and diagnosis performance.

Compared with a recent study^47^ on cancer early diagnosis that trains an SVM classifier for multi-cancer detection, our approach exploited the biological explanation of tumor-derived cfDNA fractions that exhibited significant differences between normal controls and multiple cancers, thereby providing critical biological explanations for cancer early diagnosis. Since it is easier to collect plasma samples from late-stage cancer patients than that from early-stage patients, we exploited late-stage patient data as training samples to build a cancer early diagnostic model, aiming to reduce the cost and duration of case collection. With this strategy, SRFD-Bayes was able to decipher the biological origin of tumor-derived cfDNA that could, to a large extent, correct the false negatives provided by classifiers. Therefore, although cancer samples with low tumor fractions did not appear in the training dataset, they might still be recognized by SRFD-Bayes since their estimated tumor fraction vectors were differentially distributed after deconvolution. The validation on the early-stage patients achieved the best diagnostic performance that largely outperformed the current machine learning classifiers. Due to data constraints, we only tested our diagnostic approach on public datasets, in which the number of patients with early-stage tumors was relatively limited. In the future, we can apply our approach to large-scale cancer screening, where we expect that the use of real-world data would enable promising diagnostic performance.

## Methods

### Informative methylation marker selection

Selecting informative markers from massive methylation sites is the first step for epigenetic analysis. Type-specific (TS)^9^ and type-discriminative (TD)^48^ markers are two kinds of informative markers for multi-class samples. The TS markers aim to exhibit a binary difference between one category and all other categories while the TD markers are capable of simultaneously distinguishing diverse categories, as shown in Supplementary Fig. 14a. We propose an informative marker selection approach based on matrix norm, which neither requires model training nor fits a probability distribution of each marker candidate.

Assuming that all samples are collected from *M* categories and the sample distribution of the *m*th category at a marker candidate can be characterized as a statistical histogram vector ${{{{{{\bf{a}}}}}}}_{m}\in {{\mathbb{R}}}_{+}^{B\times 1}$, in which *B* denotes the number of histogram bins. Correspondingly, the histogram features of all categories can be concatenated into a matrix ${{{{{\bf{A}}}}}}=[{{{{{{\bf{a}}}}}}}_{1},{{{{{{\bf{a}}}}}}}_{2},\ldots,{{{{{{\bf{a}}}}}}}_{M}]\in {{\mathbb{R}}}_{+}^{B\times M}(B\,\geqslant \,M \, > \,1)$. Subsequently, we define a measure to quantify the discriminability of individual marker candidate:

1 $${{{{{\mathcal{D}}}}}}=\frac{{|{{{{{\mathbf{A}}}}}}|}_{\ast }-{|{{{{{\mathbf{A}}}}}}|}_{F}}{M-\sqrt{M}}$$

where ${|\bullet|}_{\ast }$ and ${|\bullet|}_{F}$ represent the nuclear norm and Frobenius norm, separately. It can be proved (Supplementary Note 2) that the value of ${{{{{\mathcal{D}}}}}}$ falls into [0, 1]. In particular, ${{{{{\mathcal{D}}}}}}=0$ holds if and only if all the column vectors of **A** are linearly correlated, suggesting that all categories share no difference at this marker candidate, shown in Supplementary Fig. 14b. ${{{{{\mathcal{D}}}}}}=1$ holds if and only if all the column vectors of **A** are orthonormal basis. In this situation, every two categories are separately distributed and the samples in each category are highly concentrated at this marker candidate.

For each methylation site, we first judge if it could become a TS marker for category *t* by checking whether the mean methylation level of all other categories locate on the opposite side of the *t*th category. And if so, all other categories are merged into one class. Equation (1) is then adopted to calculate its binary discriminability ${{{{{{\mathcal{D}}}}}}}^{t}$. Considering that two methylation sites might share the same discriminability, we introduce the minimum distance ${d}_{{{\min }}}^{t}$ (Supplementary Fig. 14a) between the mean methylation level of category *t* and that of other categories to differentiate marker candidates. Meanwhile, we also calculate the multi-class discriminability ${{{{{{\mathcal{D}}}}}}}^{\ast }$ of this methylation site and introduce the maximal inter-class distance ${d}_{{{\max }}}^{\ast }$. Hereafter, the informative score of the marker candidate acting as *t*-TS marker and TD marker can be calculated by ${{{{{{\mathcal{S}}}}}}}_{{{{{{\rm{TS}}}}}}}^{t}={d}_{{{\min }}}^{t}{{{{{{\mathcal{D}}}}}}}^{t}$ and ${{{{{{\mathcal{S}}}}}}}_{{{{{{\rm{TD}}}}}}}^{\ast }={d}_{{{\max }}}^{\ast }{{{{{{\mathcal{D}}}}}}}^{\ast }$, respectively. Comparing ${{{{{{\mathcal{S}}}}}}}_{{{{{{\rm{TS}}}}}}}^{t}$ and ${{{{{{\mathcal{S}}}}}}}_{{{{{{\rm{TD}}}}}}}^{\ast }$, the higher one suggests an optimal marker of the methylation site and its final informative score is given by:

2 $${{{{{\mathcal{S}}}}}}=\,{{\max }}({d}_{{{\min }}}^{t}{{{{{{\mathcal{D}}}}}}}^{t},\ {d}_{{{\max }}}^{\ast }{{{{{{\mathcal{D}}}}}}}^{\ast })$$

### Semi-reference-free deconvolution (SRFD)

The methylation level of a CpG site in cancer patients’ cfDNA is substantially a linear combination of that in normal-derived and tumor-derived cfDNA (Supplementary Note 6). Supposing that the cancer patients’ cfDNA is derived from *P* types of normal sources and one tumor tissue, the methylation level of the *k*th methylation marker can be given by:

3 $${x}_{k}=\mathop{\sum }\limits_{p=1}^{P}{{{{\lambda }}}}_{p}{v}_{k,p}+\theta {u}_{k}$$

where *v*_*k,p*_ and *u*_*k*_ represent the methylation level of the *k*th marker in the *p*th normal-derived and the tumor-derived cfDNA, respectively. *λ*_*p*_ and *θ* suggest the fraction of cfDNA derived from the *p*th normal source and the tumor tissue, individually, and they are constrained by ${\sum }_{p=1}^{P}{\lambda }_{p}+\theta=1$. Combining *K* methylation markers of cfDNA, we use ${{{{{{\bf{v}}}}}}}_{1},{{{{{{\bf{v}}}}}}}_{2},\ldots,{{{{{{\bf{v}}}}}}}_{P}\in {{\mathbb{R}}}_{+}^{K\times 1}$ to denote the *K*-dimensional methylation patterns for *P* normal sources. Considering the tumor heterogeneity, we assume that the methylation signatures of the cfDNA derived from the *t*th tumor consists of *Q*_*t*_ methylation patterns and denote its *q*th pattern as ${{{{{{\mathbf{u}}}}}}}_{q}^{t}\in {{\mathbb{R}}}_{+}^{K\times 1}$. Accordingly, the vector form of Eq. (3) can be given by:

4 $${{{{{{\bf{x}}}}}}}_{i}^{0} 	=\mathop{\sum }\limits_{p=1}^{P}{\lambda }_{i,p}^{0}{{{{{{\bf{v}}}}}}}_{p},\,\forall i=1,\,2,\ldots,\,{N}_{0}\\ {{{{{{\bf{x}}}}}}}_{j}^{t} 	=\mathop{\sum }\limits_{p=1}^{P}{\lambda }_{j,p}^{t}{{{{{{\bf{v}}}}}}}_{p}+\mathop{\sum }\limits_{q=1}^{{Q}_{t}}{\theta }_{j,q}^{t}{{{{{{\bf{u}}}}}}}_{q}^{t},\,\forall j=1,\,2,\ldots,\,{N}_{t},\,t=1,\,2,\ldots,\,T$$

where ${{{{{{\bf{x}}}}}}}_{i}^{0}\in {{\mathbb{R}}}_{+}^{K\times 1}$ and ${{{{{{\bf{x}}}}}}}_{j}^{t}\in {{\mathbb{R}}}_{+}^{K\times 1}$ represent the methylation vectors of the *i*th normal sample and that of the *j*th cancer patient sample with confirmed tumor type *t*, respectively. *N*_0_ and *N*_*t*_ denote the total number of normal controls and cancer patients burdened the *t*th tumor, respectively. ${\lambda }_{i,p}^{0}$ indicates the linear combination coefficient of the *i*th normal sample with respect to the *p*th normal source and meets the constraint ${\sum }_{p=1}^{P}{\lambda }_{i,p}^{0}=1,\,\forall i=1,\,2,\ldots,\,{N}_{0}$. Similarly, ${\lambda }_{i,p}^{t}$ represents the linear combination coefficient of the *j*th sample burdened the *t*th tumor projecting on the *p*th methylation pattern of normal sources. ${\theta }_{j,q}^{t}$ suggests the tumor fraction of the *j*th sample burdened the *t*th tumor with respect to the *q*th tumor-derived cfDNA methylation pattern. ${\lambda }_{i,p}^{t}$ and ${\theta }_{j,q}^{t}$ are demanded to meet the constraint ${\sum }_{p=1}^{P}{\lambda }_{j,p}^{t}+{\sum }_{q=1}^{{Q}_{t}}{\theta }_{j,q}^{t}=1,\,\forall j=1,\,2,\ldots,\,{N}_{t}$.

The methylation vectors of both normal and cancer samples can be merged into one methylation matrix ${{{{{\bf{X}}}}}}=[{{{{{{\bf{x}}}}}}}_{1}^{0},\ldots,{{{{{{\bf{x}}}}}}}_{{N}_{0}}^{0},{{{{{{\bf{x}}}}}}}_{1}^{1},\ldots,{{{{{{\bf{x}}}}}}}_{{N}_{1}}^{1},\ldots,{{{{{{\bf{x}}}}}}}_{1}^{T},\ldots,{{{{{{\bf{x}}}}}}}_{{N}_{T}}^{T}]\in {{\mathbb{R}}}^{K\times {\sum }_{t=0}^{T}{N}_{t}}$. Correspondingly, we coalesce the normal-derived and the tumor-derived cfDNA methylation patterns **v**_*p*_ and ${{{{{{\bf{u}}}}}}}_{q}^{t}$ into one methylation pattern reference matrix ${{{{{\bf{W}}}}}}=[{{{{{{\bf{v}}}}}}}_{1},\ldots,{{{{{{\bf{v}}}}}}}_{P},{{{{{{\bf{u}}}}}}}_{1}^{1},\ldots,{{{{{{\bf{u}}}}}}}_{{Q}_{1}}^{1},\ldots,{{{{{{\bf{u}}}}}}}_{1}^{T},\ldots,{{{{{{\bf{u}}}}}}}_{{Q}_{T}}^{T}]\in {{\mathbb{R}}}^{K\times (P+{\sum }_{t=1}^{T}{Q}_{t})}$. Further, all the combination coefficients can be reshaped as a coefficient matrix ${{{{{\bf{R}}}}}}\in {{\mathbb{R}}}_{+}^{(P+{\sum }_{t=1}^{T}{Q}_{t})\times {\sum }_{t=0}^{T}{N}_{t}}$ constrained by a fixed structure ${{{{{{\mathcal{S}}}}}}}_{{{{{{\bf{R}}}}}}}$, which demands each component in the coefficient vectors of normal controls projecting on tumor patterns to be zero. Besides, in the coefficient vector of the sample burdened the *t*th tumor, each component corresponding to all other tumor patterns is demanded to be zero under the assumption that every patient suffers from only one type of cancer. The structure of each matrix is exhibited in Fig. 1b, in which white regions represent zero elements. As for a more concise representation, let $C=P+{\sum }_{t=1}^{T}{Q}_{t}$ and $N={\sum }_{t=0}^{T}{N}_{t}$, then ${{{{{\bf{X}}}}}}\in {{\mathbb{R}}}^{K\times N}$, ${{{{{\bf{W}}}}}}\in {{\mathbb{R}}}^{K\times C}$ and ${{{{{\bf{R}}}}}}\in {{\mathbb{R}}}^{C\times N}$. We can rewrite Eq. (4) as a matrix form **X** = **WR**.

As a result, the SRFD algorithm is intuitively performed to learn a methylation pattern reference **W**_†_ from the given cfDNA methylation profiles **X** consisting of normal controls and cancer patients. The mathematical description is formulated by:

5 $$\begin{array}{ll}\mathop{\min }\limits_{{{{{{\bf{W}}}}}},{{{{{\bf{R}}}}}}}&\frac{1}{2}{|{{{{{\bf{X}}}}}}-{{{{{\bf{W}}}}}}{{{{{\bf{R}}}}}}|}_{F}^{2}\\ s.t.&{{{{{{\bf{W}}}}}}}_{ij}\,\geqslant \,0,\, {{{{{{\bf{R}}}}}}}_{ij}\,\geqslant \,0,\\ &\mathop{\sum }\limits_{i=1}^{C}{{{{{{\bf{R}}}}}}}_{ij}=1\,\forall j,\\ &{{{{{{\bf{R}}}}}}}_{ij}=0\,\forall [i,\;j]\in {{{{{{\mathcal{S}}}}}}}_{{{{{{\bf{R}}}}}}}\end{array}$$

where **W**_*ij*_ ⩾ 0 and **R**_*ij*_ ⩾ 0 denote the nonnegative constraints. ${\sum }_{i=1}^{C}{{{{{{\bf{R}}}}}}}_{ij}=1\,\forall j$ demands that the sum of all coefficient vectors for one sample equals to one. ${{{{{{\bf{R}}}}}}}_{ij}=0\,\forall [i,\;j]\in {{{{{{\mathcal{S}}}}}}}_{{{{{{\bf{R}}}}}}}$ suggests that **R** is supervised by a structure ${{{{{{\mathcal{S}}}}}}}_{{{{{{\bf{R}}}}}}}$ yielded from class labels. The optimization in Eq. (5) is essentially a nonnegative matrix factorization (NMF) variant with a structural constraint^49^. In order to solve the minimization problem, we transform the structural constraint into a penalty term adding to the objective function. Besides, many studies^49,50^ report that the ${\ell }_{2,p}$-norm-based (0 < *p* < 1) minimization contributes to the robustness of the solution. Therefore, the minimization problem can be rewritten by:

6 $$\begin{array}{ll}\mathop{\min }\limits_{{{{{{\bf{W}}}}}},{{{{{\bf{R}}}}}}}&\frac{1}{2}{|{{{{{\bf{X}}}}}}-{{{{{\bf{W}}}}}}{{{{{\bf{R}}}}}}|}_{2,p}^{p}+\frac{\eta }{2}\Omega ({{{{{\bf{R}}}}}})\\ s.t.&{{{{{{\bf{W}}}}}}}_{ij}\,\geqslant \,0,\,{{{{{{\bf{R}}}}}}}_{ij}\,\geqslant \,0,\\ &\mathop{\sum }\limits_{i=1}^{C}{{{{{{\bf{R}}}}}}}_{ij}=1\,\forall j\end{array}$$

where *η* represents a constant parameter and Ω(**R**) denotes the structural penalty:

7 $$\Omega ({{{\bf{R}}}})=|{{{\bf{R}}}}\odot {{{\mathbf{{{\mathcal{M}}}}}}}_{S}|_{F}^{2}$$

where ⊙ is Hadamard product and ${{{{{{\mathbf{ {{{\mathcal{M}}}}}}}} }}}_{S}$ represents a structural binary mask, in which the positions of 1 are derived from the structural constraint ${{{{{{\mathcal{S}}}}}}}_{{{{{{\bf{R}}}}}}}$. We introduce Lagrange multiplier **Ψ** and **Φ** for the matrix factors **W** and **R** due to their nonnegative constraints. Correspondingly, a Lagrange function can be formulated by:

8 $${\mathcal L}=\frac{1}{2}{|{{{{{\bf{X}}}}}}-{{{{{\bf{W}}}}}}{{{{{\bf{R}}}}}}|}_{2,p}^{p}+\frac{\eta }{2}{|{{{{{\bf{R}}}}}}\odot {{{{{{\mathbf{ {{{\mathcal{M}}}}}}}} }}}_{S}|}_{F}^{2}+{{{{{\rm{Tr}}}}}}({{{{{\bf{\Psi }}}}}}{{{{{{\bf{W}}}}}}}^{T})+{{{{{\rm{Tr}}}}}}({{{{{\bf{\Phi }}}}}}{{{{{{\bf{R}}}}}}}^{T})$$

The partial derivatives of ${\mathcal L}$ with respect to the matrix factors **W** and **R** can be calculated by:

9 $$\frac{\partial {\mathcal L} }{\partial {{{{{\bf{W}}}}}}} \, 	=({{{{{\bf{W}}}}}}{{{{{\bf{R}}}}}}{{{{{\bf{D}}}}}}{{{{{{\bf{R}}}}}}}^{T}-{{{{{\bf{X}}}}}}{{{{{\bf{D}}}}}}{{{{{{\bf{R}}}}}}}^{T})+{{{\bf{\Psi }}}}=0\\ \frac{\partial {\mathcal L} }{\partial {{{{{\bf{R}}}}}}} \, 	={{{{{{\bf{W}}}}}}}^{T}({{{{{\bf{W}}}}}}{{{{{\bf{R}}}}}}-{{{{{\bf{X}}}}}}){{\bf{D}}}+\eta ({{{{{\bf{R}}}}}}\odot {{{{{{\bf{ {\mathcal M}}}}} }}}_{\it{S}})+{{{\bf{\Phi }}}}=0$$

where **D** is a diagonal matrix with each diagonal entry as ${{{{{{\bf{D}}}}}}}_{kk}=\frac{p}{2{|{{{{{{\bf{z}}}}}}}_{k}|}_{2}^{2-p}}$, **Z** = **X**-**WR**. Using the Kuhn-Tucker condition **Ψ**_*ih*_ **W**_*ih*_ = 0 and **Φ**_h*j*_ **R**_*hj*_ = 0, we have:

10 $$\begin{array}{c}{({{{{{\bf{W}}}}}}{{{{{\bf{R}}}}}}{{{{{\bf{D}}}}}}{{{{{{\bf{R}}}}}}}^{T})}_{ih}{{{{{{\bf{W}}}}}}}_{ih}-{({{{{{\bf{X}}}}}}{{{{{\bf{D}}}}}}{{{{{{\bf{R}}}}}}}^{T})}_{ih}{{{{{{\bf{W}}}}}}}_{ih}=0\\ {({{{{{{\bf{W}}}}}}}^{T}{{{{{\bf{W}}}}}}{{{{{\bf{R}}}}}}{{{{{\bf{D}}}}}}-{{{{{{\bf{W}}}}}}}^{T}{{{{{\bf{X}}}}}}{{{{{\bf{D}}}}}})}_{hj}{{{{{{\bf{R}}}}}}}_{hj}+\eta {({{{{{\bf{R}}}}}}\odot {{{{{{\bf{ {\mathcal M}}}}} }}}_{S})}_{hj}{{{{{{\bf{R}}}}}}}_{hj}=0\end{array}$$

The corresponding updating rule for **W** and **R** can be given by:

11 $$\begin{array}{c}{{{{{{\bf{W}}}}}}}_{ih}\leftarrow {{{{{{\bf{W}}}}}}}_{ih}\frac{{[{{{{{\bf{X}}}}}}{{{{{\bf{D}}}}}}{{{{{{\bf{R}}}}}}}^{T}]}_{ih}}{{[{{{{{\bf{W}}}}}}{{{{{\bf{R}}}}}}{{{{{\bf{D}}}}}}{{{{{{\bf{R}}}}}}}^{T}]}_{ih}}\\ {{{{{{\bf{R}}}}}}}_{hj}\leftarrow {{{{{{\bf{R}}}}}}}_{hj}\frac{{[{{{{{{\bf{W}}}}}}}^{T}{{{{{\bf{X}}}}}}{{{{{\bf{D}}}}}}]}_{hj}}{{[{{{{{{\bf{W}}}}}}}^{T}{{{{{\bf{W}}}}}}{{{{{\bf{R}}}}}}{{{{{\bf{D}}}}}}]}_{hj}+\eta {[{{{{{\bf{R}}}}}}\odot {{{{{{\bf{ {\mathcal M}}}}} }}}_{S}]}_{hj}}\end{array}$$

Subsequently, the iterative algorithm for SRFD is briefly described in Supplementary Box 1.

With the learned optimal methylation reference **W**_†_, the fraction vector **h** of a test cfDNA methylation profile ${{{{{\bf{x}}}}}}\in {{\mathbb{R}}}_{+}^{K\times 1}$ can be predicted by:

12 $$\begin{array}{ll}\mathop{\min }\limits_{{{{{{\bf{h}}}}}}}&\frac{1}{2}{|{{{{{\bf{x}}}}}}-{{{{{{\bf{W}}}}}}}_{{{\dagger}} }{{{{{\bf{h}}}}}}|}^{2}\\ s.t.&\,{{{{{{\bf{h}}}}}}}_{i}\,\geqslant \,0,\mathop{\sum }\limits_{i=1}^{C}{{{{{{\bf{h}}}}}}}_{i}=1\end{array}$$

The first *P* components in **h** represent the fractions of normal sources while the rest components indicate the fractions of *T* tumor sources. In our simulation experiments, the deconvolution performance was evaluated in two respects, source fraction and tumor fraction. The former separately sums the corresponding source components of the specific tumor while the latter sums all tumor components in the predicted fraction vectors.

### Bayesian diagnostic model based on estimated tumor fractions (SRFD-Bayes)

For a test cfDNA sample **x** and its estimated fraction vector **h**, we first extract all tumor components of **h** to shape a predicted tumor fraction vector $\hat{{{{{{\bf{\uptheta }}}}}}}$. Inherently, the cancer diagnostic decision can be made by maximizing the posterior probability:

13 $$\mathop{\max }\limits_{y\in {{{{{\mathcal{Y}}}}}}}p(y|{{{{{\bf{x}}}}}},\hat{{{{{{\bf{\uptheta }}}}}}})$$

where ${{{{{\mathcal{Y}}}}}}$ represents the label set. According to Bayes’ theorem, we have:

14 $$p(y|{{{{{\bf{x}}}}}},\hat{{{{{{\bf{\uptheta }}}}}}})=\frac{p(\hat{{{{{{\bf{\uptheta }}}}}}}|y,\, {{{{{\bf{x}}}}}})p(y|{{{{{\bf{x}}}}}})p({{{{{\bf{x}}}}}})}{p(\hat{{{{{{\bf{\uptheta }}}}}}}|{{{{{\bf{x}}}}}})p({{{{{\bf{x}}}}}})}=\frac{p(\hat{{{{{{\bf{\uptheta }}}}}}}|y,\, {{{{{\bf{x}}}}}})p(y|{{{{{\bf{x}}}}}})}{p(\hat{{{{{{\bf{\uptheta }}}}}}}|{{{{{\bf{x}}}}}})}$$

Since $p(\hat{{{{{{\bf{\uptheta }}}}}}}|{{{{{\bf{x}}}}}})$ is irrelevant to the labels, Eq. (14) can be simplified as:

15 $$\begin{array}{c}p(y|{{{{{\bf{x}}}}}},\, \hat{{{{{{\bf{\uptheta }}}}}}})\propto p(\hat{{{{{{\bf{\uptheta }}}}}}}|y,\, {{{{{\bf{x}}}}}})p(y|{{{{{\bf{x}}}}}})\end{array}$$

where $p(\hat{{{{{{\bf{\uptheta }}}}}}}|y,{{{{{\bf{x}}}}}})$ suggests the conditional probability distribution of $\hat{{{{{{\bf{\uptheta }}}}}}}$ with the given label *y* and methylation data **x**. *p*(*y*|**x**) denotes the probability distribution of the diagnostic decision without the guidance of $\hat{{{{{{\bf{\uptheta }}}}}}}$, which acts as the prior of the Bayesian inference and can be obtained by training ordinary classifiers (SVM in this study) on the original methylation profiles. Assuming that each component of $\hat{{{{{{\bf{\uptheta }}}}}}}$ is independent and conforms a Beta distribution, correspondingly, Eq. (15) can be formulated by:

16 $$\begin{array}{c}p(y|{{{{{\bf{x}}}}}},\hat{{{{{{\bf{\uptheta }}}}}}}) \propto {\prod }_{i}p({\hat{{{{{{\bf{\uptheta }}}}}}}}_{i}|y,{{{{{\bf{x}}}}}})p(y|{{{{{\bf{x}}}}}})={\prod }_{i}{{{{{\rm{Beta}}}}}}({\alpha }_{i},{\beta }_{i})p(y|{{{{{\bf{x}}}}}})\end{array}$$

where ${{{{{\rm{Beta}}}}}}({\alpha }_{i},{\beta }_{i})$ denotes the Beta distribution of ${\hat{{{{{{\bf{\uptheta }}}}}}}}_{i}$ with parameters *α*_*i*_ and *β*_*i*_, which can be estimated from the tumor fraction vectors of the training samples.

With respect to the training process of SRFD-Bayes, the input of classifiers is the original methylation features of each training sample, which is a *K*-dimensional vector with each component representing the methylation level of a methylation site. By employing the reference database **W**_†_ estimated by SRFD, a $C$-dimensional fraction vector of each training sample is obtained, where the tumor components are extracted to establish the conditional Beta distribution. SRFD-Bayes is implemented and performed using MATLAB R2018b with Parallel Computing Toolbox 6.13, Statistics and Machine Learning Toolbox 11.4, and Deep Learning Toolbox 12.0.

### Synthetic minority oversampling

Synthetic minority oversampling is utilized to achieve the balance among different categories in the model training procedure. Instead of direct oversampling on the original methylation signatures, we adopt borderline-SMOTE algorithm^51^ to synthesize a new fraction vector ${\tilde{{{{{{\bf{h}}}}}}}}_{i}$ for the minority category *i*. Afterwards, the convolution ${{{{{{\bf{W}}}}}}}_{{{\dagger}} }{\tilde{{{{{{\bf{h}}}}}}}}_{i}+{{{{{{\bf{\upvarepsilon }}}}}}}_{i}$ is employed to generate a new synthesized methylation profile, where **W**_†_ denotes the learned reference database. **ε**_i_ suggests a reconstruction error vector that is randomly sampled from a normal distribution fitted by the reconstruction errors in the semi-reference-free deconvolution.

### Statistics & reproducibility

All the data used in this paper are collected from publicly accessible datasets. No statistical method was used to predetermine sample size. No data were excluded from the analyses. The experiments were not randomized. The Investigators were not blinded to allocation during experiments and outcome assessment.

### Reporting summary

Further information on research design is available in the Nature Portfolio Reporting Summary linked to this article.

## Supplementary information

Supplementary information

Reporting Summary

## Data Availability

The main data supporting the results are available within this article as well as its Supplementary information. All the datasets adopted in this study are publicly available. The cfDNA-SNV data analyzed in this study were collected from Lung-CLiP^52^ [https://clip.stanford.edu/]. The DNA methylation profiles of tumor tissues adopted in this study were collected from TCGA [https://portal.gdc.cancer.gov/]. The normal blood cfDNA methylation profiles were collected from GSE40279. The plasma cfDNA methylation profiles were collected from GSE122126, GSE108462, GSE129374 and the repository NCOMMS-20-10056-T on GitHub [https://github.com/ncomms-20-10056-t/ncomms-20-10056-t]. The plasma cfDNA with 10 methylation sites for HCC patients and normal controls were collected from the Supplementary materials of the previous study^31^ [https://www.nature.com/articles/nmat4997#Sec19]. Source data are provided with this paper.

## Source data

Source data

## Code availability

The code of our approach is available on GitHub in the repository SRFD-Bayes (https://github.com/Astaxanthin/SRFD-Bayes) and a Zenodo repository 10.5281/zenodo.7357786.
