# Publisher Correction: Tumor fractions deciphered from circulating cell-free DNA methylation for cancer early diagnosis

**DOI:** 10.1038/s41467-023-36010-4

**Published:** 2023-01-19

**Authors:** Xiao Zhou, Zhen Cheng, Mingyu Dong, Qi Liu, Weiyang Yang, Min Liu, Junzhang Tian, Weibin Cheng

**Affiliations:** 1 Department of Automation, Tsinghua University, Beijing, 100084 China; 2 Institute for Healthcare Artificial Intelligence Application, Guangdong Second Provincial General Hospital, Guangzhou, 510317 China

**Keywords:** Machine learning, Diagnostic markers, Cancer

Correction to: *Nature Communications* 10.1038/s41467-022-35320-3, published online 13 December 2022

The original version of this manuscript contained a link to the Peer Review file which the authors had opted-out of for publication. This Peer Review file has now been redacted. In addition, the ‘Peer Review Information’ statement has been updated to remove the text “Peer reviewer reports are available.”

