# Supplementary material for: Tumor fractions deciphered from circulating cell-free DNA methylation for cancer early diagnosis
Source: Nat Commun. 2022 Dec 13;13:7694. doi: 10.1038/s41467-022-35320-3 (PMC9744803; doi:10.1038/s41467-022-35320-3)
Supplement: Supplementary file 1 — Supplementary information [file 41467_2022_35320_MOESM1_ESM.pdf]

Tumor fractions deciphered from circulating cell-free DNA methylation for cancer early diagnosis

Xiao Zhou[1,3], Zhen Cheng[1,3], Mingyu Dong[1], Qi Liu[1], Weiyang Yang[1], Min Liu[1,2✉], Junzhang Tian[2✉] and Weibin Cheng[2✉]

[1]Department of Automation, Tsinghua University, Beijing, China

[2]Institute for Healthcare Artificial Intelligence Application, Guangdong Second Provincial General Hospital, Guangzhou, 510317, China

[3]These authors contributed equally: Xiao Zhou, Zhen Cheng.

✉Corresponding authors: Min Liu, Junzhang Tian and Weibin Cheng.

Email: lium@mail.tsinghua.edu.cn; jz.tian@163.com; chwb817@gmail.com;

Table of contents

Supplementary Note 1. The number of patients carrying tumor-derived mutations among different cancer stages.

Supplementary Note 2. The proposition and the corresponding proof about the informative score.

Supplementary Note 3. Details for the generation of simulation dataset.

Supplementary Note 4. Parameter configuration of semi-reference-free deconvolution (SRFD).

Supplementary Note 5. Implementation details for tissue-requiring approaches and machine learning classifiers.

Supplementary Note 6. The calculation of the methylation level of a CpG site in cancer patients' cfDNA.

Supplementary Box 1. The iterative algorithm for SRFD.

Supplementary Table 1. Dataset split on the samples of normal controls and tumor tissues.

Supplementary Table 2. The probabilities of each copy number for 10%, 30% and 50% copy number variation (CNV) events.

Supplementary Table 3. The quantitative distribution of simulation plasma samples for each category used in different experiments.

Supplementary Table 4. The number of available samples in each real dataset that adopted in this study.

Supplementary Fig. 1. The statistics of non-small cell lung cancer (NSCLC) patients with tumor-derived mutations in Lung-CLiP[1].

Supplementary Fig. 2. Visualization for the generation of simulated plasma data.

Supplementary Fig. 3. Top-1 TS methylation markers for different tumor type, including Breast Invasive Carcinoma (BRCA), Colon Adenocarcinoma (COAD), Lung Squamous Cell Carcinoma & Lung Adenocarcinoma (LUNG) and Liver Hepatocellular Carcinoma (LIHC).

Supplementary Fig. 4. Experimental results on parameter configuration.

Supplementary Fig. 5. Experimental results on parameter study of CancerLocator.

Supplementary Fig. 6. Comparison of overall deconvolution performance on simulation datasets with 10% CNV events and 50% CNV events.

Supplementary Fig. 7. The comparison of detailed deconvolution performance achieved by different approaches on simulation tumor samples with 10% CNV events and 50% CNV events.

Supplementary Fig. 8. Comparison of localization performance on cancer samples with tumor fraction more than 0.1.

Supplementary Fig. 9. AUC performance comparison on patient cfDNA when using different number of samples and markers for training of SRFD.

Supplementary Fig. 10. AUC performance comparison of distinguishing cancer patients from normal controls when using different number of samples for training of SRFD and SRFD-Bayes.

Supplementary Fig. 11. Diagnostic performance on pre-diagnosis patients.

Supplementary Fig. 12. Diagnostic results before and after synthetic oversampling strategy.

Supplementary Fig. 13. The comparison of diagnostic performance among different approaches.

Supplementary Fig. 14. The schematic diagram of TS and TD markers as well as the visualization of the discriminability.

Supplementary Note 1. The number of patients carrying tumor-derived mutations among different cancer stages.

Employing tumor-derived single nucleotide variation (SNV), Chabon et al.[1] proposed a semi-supervised machine learning model to predict the source of circulating cell-free DNA (cfDNA) and then applied the model for NSCLC detection. After analyzing their public data, we found that the SNVs in plasma cfDNA detected from 51 (70 in total) cancer patients did not overlap with the mutations detected from their corresponding tumor tissues. Besides, 39 cases out of the 51 non-overlap patients were diagnosed as early stages (IA, IB), in contrast, the tumor-derived mutations are found from about 61% (14/23) of patients with later-stage tumors (IIB, IIIA, IIIB), which is exhibited in Supplementary Fig. 1. This study suggested a persuasive phenomenon that whether the tumor-derived mutations are detectable in plasma cfDNA is highly dependent on the cancer stages.

Supplementary Note 2. The proposition and the corresponding proof about the informative score.

To identify TS and TD markers, some studies[2] calculate simple statistical characteristics, such as mean and standard, of each class. Smyth et al.[3] manage to find differentially methylated probes (DMP) with statistical significance between two groups. Xia et al.[4] adopt the area under the curve (AUC) to measure the discriminability of each methylation site between two classes. Recourse to machine learning skills, many studies[5,6] rank marker candidates according to their contribution to the classification assignments. However, all above approaches fail to uniformly excavate and evaluate both TS and TD markers. In this paper, we propose a novel approach based on matrix norm to select informative markers, which neither requires model training nor fits a probability distribution of each marker candidate. A measure to quantify the discriminability of the marker candidate is defined in Eq. (1) and three corresponding propositions are presented as follows.

Proposition 1. Given $\mathbf{A} \in \mathbb{R}_{+}^{B\times M}(B \geq M > 1)$, $\sum_{j=1}^{B}\mathbf{A}_{ij} = 1$, and $\mathcal{D} = \frac{\|\mathbf{A}\|_{*}-\|\mathbf{A}\|_{F}}{M-\sqrt{M}}$, then $\mathcal{D} \in [0,1]$.

Proof. According to the equivalence of matrix norms, the inequality $\|\mathbf{A}\|_{F} \leq \|\mathbf{A}\|_{*} \leq \sqrt{M}\|\mathbf{A}\|_{F}$ holds, which results in $0 \leq \mathcal{D} \leq \frac{\|\mathbf{A}\|_{F}(\sqrt{M}-1)}{M-\sqrt{M}} = \frac{\|\mathbf{A}\|_{F}}{\sqrt{M}}$. Since the entries in $\mathbf{A}$ are nonnegative and restricted by $\sum_{j=1}^{B}\mathbf{A}_{ij} = 1$, we have $\|\mathbf{A}\|_{F}^{2} = \sum_{i=1}^{M}\sum_{j=1}^{B}\mathbf{A}_{ij}^{2} \leq \sum_{i=1}^{M}\left(\sum_{j=1}^{B}\mathbf{A}_{ij}\right)^{2} = M$. The first equality and the second inequality hold according to the definition of F-norm and the inequality of arithmetic and geometric means (AM-GM), respectively. As a result, $\|\mathbf{A}\|_{F} \leq \sqrt{M}$, such that $0 \leq \mathcal{D} \leq 1$. □

Proposition 2. $\mathcal{D} = 0$ holds if and only if $\text{rank}(\mathbf{A}) = 1$, *i.e.* all the column vectors of $\mathbf{A}$ are linearly correlated.

Proof. According to the definition of nuclear norm, we have $\|\mathbf{A}\|_{*} = \sum_{i=1}^{M}\sigma_{i}$, where σ_{i} suggest the ith largest singular value of $\mathbf{A}$.

Sufficiency: $\text{rank}(\mathbf{A}) = 1$ suggests $\sigma_i = 0$ for $i = 2,3, \dots, M$. Therefore, $\sum_{i=1}^{M} \sigma_i = \sqrt{\sum_{i=1}^{M} \sigma_i^2}$, *i.e.* $||\mathbf{A}||_* = ||\mathbf{A}||_F$, such that $\mathcal{D} = 0$.

Necessity: $\mathcal{D} = 0$ indicates $||\mathbf{A}||_* = ||\mathbf{A}||_F$, such that $\sum_{i=1}^{M} \sigma_i = \sqrt{\sum_{i=1}^{M} \sigma_i^2}$, *i.e.* $\left(\sum_{i=1}^{M} \sigma_i\right)^2 = \sum_{i=1}^{M} \sigma_i^2$. Expanding the equality, we know that the sum of all cross terms among singular values has to be zero, *i.e.* $\sum_{i=1}^{M-1} \sum_{j=i+1}^{M} \sigma_i \sigma_j = 0$. Now we suppose that there exist at least two nonzero singular values σ_p and σ_q. And then their cross term meets $\sigma_p \sigma_q > 0$ owing to the non-negativity of singular values, such that $\sum_{i=1}^{M-1} \sum_{j=i+1}^{M} \sigma_i \sigma_j > 0$, which contradicts $\sum_{i=1}^{M-1} \sum_{j=i+1}^{M} \sigma_i \sigma_j = 0$. Therefore, there is only one nonzero singular value, which leads to $\text{rank}(\mathbf{A}) = 1$, *i.e.* all column vectors of $\mathbf{A}$ are linearly correlated. □

Proposition 3. $\mathcal{D} = 1$ holds if and only if all the column vectors of $\mathbf{A}$ are orthonormal basis.

Proof. Let the singular-value decomposition of $\mathbf{A}$ be $\mathbf{A} = \mathbf{UDV}^T$.

Sufficiency: Since $\mathbf{A}$ is constructed by orthonormal basis, we have $\mathbf{A}^T\mathbf{A} = \mathbf{I}$, such that $\mathbf{A}^T\mathbf{A} = \mathbf{VD}^T\mathbf{U}^T\mathbf{UDV}^T = \mathbf{VD}^T\mathbf{DV}^T = \mathbf{I}$, thus $\mathbf{D}^T\mathbf{D} = \mathbf{I}$. As $\mathbf{D}$ is a diagonal matrix formed by singular values, we have $\sigma_1 = \sigma_2 = \cdots = \sigma_M = 1$, such that $||\mathbf{A}||_F = \sqrt{M}$ and $||\mathbf{A}||_* = M$, which result in $\mathcal{D} = 1$.

Necessity: According to Proposition 1, $\mathcal{D} = 1$ suggests $\frac{||\mathbf{A}||_* - ||\mathbf{A}||_F}{M - \sqrt{M}} = \frac{||\mathbf{A}||_F}{\sqrt{M}} = 1$, *i.e.* $||\mathbf{A}||_* = \sqrt{M}||\mathbf{A}||_F$ and $||\mathbf{A}||_F = \sqrt{M}$. Therefore, $\sum_{i=1}^{M} \sigma_i = \sqrt{M \sum_{i=1}^{M} \sigma_i^2} = M$. According to the AM-GM inequality, we have $\sigma_1 = \sigma_2 = \cdots = \sigma_M = 1$, such that $\mathbf{D}^T\mathbf{D} = \mathbf{I}$ and thus $\mathbf{A}^T\mathbf{A} = \mathbf{I}$, *i.e.* the column vectors of $\mathbf{A}$ are orthogonal. From Proposition 1, we know $||\mathbf{A}||_F^2 = \sum_{i=1}^{M} \sum_{j=1}^{B} \mathbf{A}_{ij}^2 \le \sum_{i=1}^{M} \left(\sum_{j=1}^{B} \mathbf{A}_{ij}\right)^2 = M$. As a result, $||\mathbf{A}||_F = \sqrt{M}$ leads to $\sum_{j=1}^{B} \mathbf{A}_{ij}^2 = \left(\sum_{j=1}^{B} \mathbf{A}_{ij}\right)^2$. Due to the non-negative property of the entries in $\mathbf{A}$ and the restriction $\sum_{j=1}^{B} \mathbf{A}_{ij} = 1$, each column of $\mathbf{A}$ has one and only one nonzero element, whose value is 1. In this situation, all the column vectors of $\mathbf{A}$ are orthonormal basis.

□

Supplementary Note 3. Details for the generation of simulation dataset.

To quantify the performance of tumor fraction prediction, we collected 656 normal blood DNA methylation profiles from GSE40279[7] and DNA methylation data from 5 types of solid tumor tissues, including 775 BRCA, 293 COAD, 821 LUNG, 375 LIHC, 484 PRAD from The Cancer Genome Atlas (TCGA) to generate simulated methylation profiles of plasma cfDNA from cancer patients. Each class is randomly divided into a training, a validation and a test dataset by a ratio of 4:1:5, which is shown in Supplementary Table 1. To simulate normal plasma cfDNA more realistically, we employed 8 normal plasma cfDNA samples from GSE122126[8], then compared them with the normal blood DNA methylation profiles and excluded the sites exhibiting significant differences (p<0.05) between these two datasets. The strategy to yield mixed simulation samples of cancer patients in this paper brings into correspondence with CancerLocator[9] for comparison. Briefly, each training plasma sample of tumor type t is generated by linearly combining one normal blood sample and one tumor tissue sample, which are randomly chosen from the training dataset of the normal control and the tth tumor, respectively. Considering the copy number variant (CNV) events, the methylation level of the kth marker on each synthetic methylation vector is given by:

$$\mathbf{x}_k = (1-\theta'_k)\mathbf{v}_k + \theta'_k \mathbf{u}_k$$
$$\theta'_k = \frac{\theta c_k}{\theta c_k + 2(1-\theta)} \qquad \text{(S1)}$$

where $\mathbf{v}_k$ and $\mathbf{u}_k$ denote the methylation level on the kth marker of normal cfDNA and tumor tissue DNA methylation features. θ and θ'_k represent the tumor fraction before and after taking into account of the CNV events, c_k denotes the copy number of tumor-derived cfDNA. The copy number of each CpG site in normal sources is fixed to 2 while that in tumor sources varies from zero to five, i.e., $c_k \in \{0,1,2,3,4,5\}$. Consistent with CancerLocator, we quantified the pre-defined probability of CNV events as 10%, 30% and 50%, individually. The probabilities of each copy number are configured in Supplementary Table 2. θ is set to be uniformly distributed in a fixed range from 0 to 0.5 with a length of 0.05, *i.e.* $\{\theta \in \mathrm{U}\big(0.05s, 0.05(s+1)\big)\ \big|s = 0,1,\dots,9\}$. The validation/test simulation samples are generated from the test tissue/plasma data using the same procedure described above. The visualization for the generation of simulated plasma data is shown in Supplementary Fig. 2.

Supplementary Note 4. Parameter configuration of semi-reference-free deconvolution (SRFD).

The norm parameter $p\ (0 < p < 1)$ and the coefficient of the structural penalty η in Supplementary Algorithm 1 are set to 0.5 and 1000 across all experiments in this study. The maximum number of iterations for SRFD is set to 1000.

We designed a parameter study (Supplementary Fig. 4). The number of markers was set 2~500 per category while the number of normal and tumor patterns were set to 1~5 and 1~9, respectively. Due to the various normal sources in plasma, the number of normal patterns was set to lager than that of tumor patterns. It can be concluded that the best performance on validation dataset was achieved when using Top-50 markers, 7 normal patterns and 2 tumor patterns. Although the RMSE might still be reduced with a larger number of markers and corresponding tumor/normal patterns, the experimental cost for the potential clinical study and large-scale applications is positively correlated with the number of markers. It is necessary to find the minimal number of discriminative markers while achieving satisfactory performance. Based on this principle, we had chosen Top-50 markers for each category.

Correspondingly, we configured the number of normal and tumor patterns as 7 and 2 throughout all experiments in this study. The marker number of methylations (Top-50), and plasma sample number (400 for each category) were consistent throughout most experiments (including simulation dataset, GSE108462[10] and GSE129374[11] real datasets). Since other cfDNA datasets (223 cancer patients in Chen et al.[12] and 1050 HCC patients in Xu et al. [13]) contained new methylation sites/regions, the SRFD-Bayes were independently trained with corresponding sites/regions (10 and 595, respectively).

Supplementary Note 5. Implementation details for tissue-requiring approaches and machine learning classifiers

The deconvolution and diagnostic strategy of CancerLocator is set to the same as the original study. There was a critical parameter in CancerLocator, which acted as a tumor fraction threshold for distinguishing cancer patients from normal controls. This parameter was pre-set to 0.01 in the source code. We implemented a parameter study on the tumor fraction threshold to achieve its best performance in simulation dataset. The experimental results, shown in Supplementary Fig. 5, suggested that the best average localization performance of approximately 0.89 was achieved at a threshold of 0.1. Correspondingly, all the experiments of CancerLocator in the main context were implemented with the threshold of 0.1.

The reference database for the NNLS approach is generated using the average DNA methylation over biological replicates in each category.

The machine learning classifiers, including random forest (RF), multilayer perception (MLP) and support vector machine (SVM), are implemented and trained in MATLAB. The number of trees in RF is set to 100. Linear kernel function is adopted in SVM. The MLP is constructed by a three-layer perceptron, in which the hidden layer has 10 neurons.

Supplementary Note 6. The calculation of the methylation level of a CpG site in cancer patients' cfDNA

The methylation of a CpG site k in circulating cfDNA is quantified by the fraction of methylated cytosines among the total cytosines. Without loss of generality, we assume that there are M_k normal-derived cfDNA and N_k tumor-derived cfDNA that together covers the kth CpG site in a cancer patient's plasma, where the cytosines from M_k^1 $(M_k^1 < M_k)$ normal-derived cfDNA and $N_k^1 (N_k^1 < N_\mathrm{k})$ tumor-derived cfDNA are methylated. Accordingly, the methylation level of the CpG site k in the cancer patient's cfDNA, x_k, can be calculated by:

$$x_k = \frac{M_k^1 + N_k^1}{M_k + N_k} = \frac{M_k}{M_k + N_k}\frac{M_k^1}{M_k} + \frac{N_k}{M_k + N_k}\frac{N_k^1}{N_k} \qquad (S2)$$

where $\frac{M_k}{M_k+N_k}$ and $\frac{N_k}{M_k+N_k}$ indicate the fraction of normal-derived and tumor-derived cfDNA, respectively. According to the definition of the methylation level, $\frac{M_k^1}{M_k}$ and $\frac{N_k^1}{N_k}$ represent the methylation levels of normal-derived and tumor-derived cfDNA on the kth CpG site, separately. For convenient of description, let $\theta = \frac{N_k}{M_k+N_k}$, $v_k = \frac{M_k^1}{M_k}$, $u_k = \frac{N_k^1}{N_k}$. The Equation (2) can be rewritten as $x_k = (1-\theta)v_k + \theta u_k$.

Assuming that the cancer patients' cfDNA is derived from P types of normal sources and one tumor tissue, the methylation level of the kth methylation marker can be given by:

$$x_k = \sum_{p=1}^{P} \lambda_p \, v_{k,p} + \theta u_k \qquad (S3)$$

where $v_{k,p}$ represent the methylation level of the kth marker in the pth normal-derived. λ_p and θ suggest the fraction of cfDNA derived from the pth normal source and the tumor tissue, individually, and they are constrained by $\sum_{p=1}^{P} \lambda_p + \theta = 1$.

Supplementary Box 1 ▌ The iterative algorithm for SRFD

1. **Input:**

Methylation profiles $\mathbf{X} \in \mathbb{R}_{+}^{K\times N}$

Structural binary mask $\boldsymbol{\mathcal{M}}_S$

Parameters: $1 \leq C \leq \min(K, N)$, p, η, Convergence threshold ε and Maximum iterations T

2. **Initialize:**

$t = 0$, $\mathbf{D} = \mathbf{I}$. Randomly initialize the methylation pattern reference matrix and coefficient matrix $\mathbf{W}_t$, $\mathbf{R}_t$.

3. **Repeat:** repeat the following steps until the convergence achieves.

While $t < T$ **do**

3a. Compute the reconstruction error:

$$\mathbf{Z} = \mathbf{X} - \mathbf{W}_t\mathbf{R}_t$$

$$\mathbf{D}_{kk} = \frac{p}{2\|\boldsymbol{z}_k\|_2^{2-p}} \ \forall k \in \{1,2,\dots,R\}$$

3b. Update the methylation pattern reference matrix and coefficient matrix:

$$\mathbf{W}_{t+1}^{ih} \leftarrow \mathbf{W}_t^{ih} \frac{[\mathbf{XDR}_t^T]_{ih}}{[\mathbf{W}_t\mathbf{R}_t\mathbf{DR}_t^T]_{ih}}$$

$$\mathbf{R}_{t+1}^{hj} \leftarrow \mathbf{R}_t^{hj} \frac{[\mathbf{W}_{t+1}^T\mathbf{XD}]_{hj}}{[\mathbf{W}_{t+1}^T\mathbf{W}_{t+1}\mathbf{R}_t\mathbf{D}]_{hj} + \eta[\mathbf{R}_t \odot \boldsymbol{\mathcal{M}}_S]_{hj}}$$

3c. Normalize the coefficient matrix:

$$\mathbf{R}_{t+1} = \mathrm{Normalize}(\mathbf{R}_{t+1})$$

3d. Check for convergence:

if $\mathrm{abs}\big(\mathrm{Err}(\mathbf{W}_{t+1}, \mathbf{R}_{t+1}), \mathrm{Err}(\mathbf{W}_t, \mathbf{R}_t)\big) < \varepsilon$

break

end

$t = t + 1$

end

4. **Output:**

Converged $\mathbf{W} \in \mathbb{R}_{+}^{K\times C}$ and $\mathbf{R} \in \mathbb{R}_{+}^{C\times N}$

Supplementary Table 1. Dataset splits on the samples of normal controls and tumor tissues.

Category	Normal	BRCA	COAD	LUNG	LIHC	PRAD
All	656	775	293	821	375	484
Train	263	311	118	329	151	194
Validate	65	77	29	82	37	48
Test	328	387	146	410	187	242

Supplementary Table 2. The probabilities of each copy number for 10%, 30% and 50% copy number variation (CNV) events.

c_k	0	1	2	3	4	5
CNV-10%	0.002	0.053	0.9	0.035	0.008	0.002
CNV-30%	0.005	0.16	0.7	0.105	0.025	0.005
CNV-50%	0.008	0.266	0.5	0.178	0.04	0.008

Supplementary Table 3. The quantitative distribution of simulation plasma samples for each category used in different experiments. θ_T denotes tumor fraction while sample number of each tumor (five types) is equal.

Experiments	Dataset	Normal	Each Tumor	All
Deconvolution	Train	400	400	2,400
	Validation	100	100	600
Diagnosis A	Train (all tumor fractions)	200	40	400
Diagnosis B	Train ($\theta_T > 0.1$)	200	32	360
Deconvolution<br>Diagnosis A & B	Test	400	400	2,400

Supplementary Table 4. The quantitative distribution of available samples in each real dataset that is adopted in this study.

Name	Journal	Year	Accession #	Data type	# Samples	# Sites/Regions
Hannum et al. [7]	Molecular Cell	2013	GSE40279	Blood DNA	656 Normal	450K
Moss et al. [8]	Nature Communications	2018	GSE122126	cfDNA	12 Normal	450K
Gordevičius et al. [10]	Clinical Cancer Research	2018	GSE108462	cfDNA	29 Prostate	450K
Hlady et al. [11]	Theranostics	2019	GSE129374	cfDNA	22 HCC &Cirrhosis<br>21 Cirrhosis	450K
Xu et al.[13]	Nature Materials	2017	Supplement	cfDNA	835 normal<br>1,050 HCC	10
Chen et al.[12]	Nature Communications	2020	Supplement	cfDNA	414 normal<br>7 Colorectal<br>56 Lung<br>23 Liver<br>68 Esophageal<br>69 Stomach	595

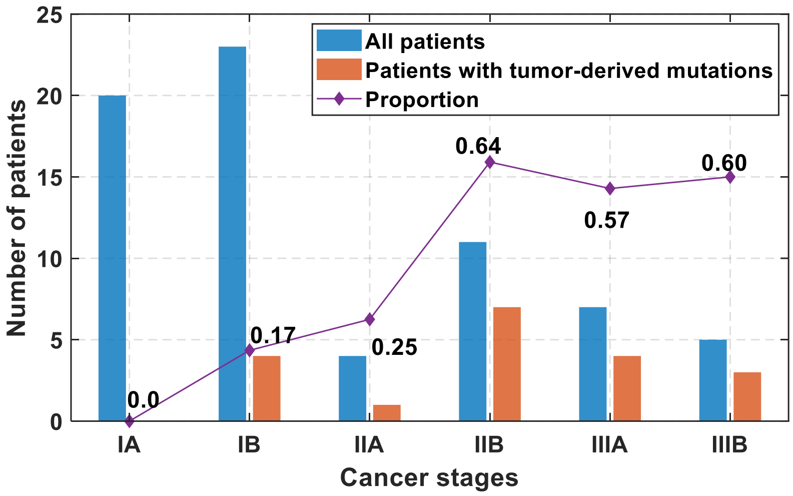

Supplementary Fig. 1 The statistics of NSCLC patients with tumor-derived mutations in Lung-CLiP[1]. The proportion (purple) in the graph is calculated in each stage of cancer according to the number of patients with tumor-derived mutations (red) dividing the total number of NSCLC patients (blue). Source data are provided as a Source Data file.

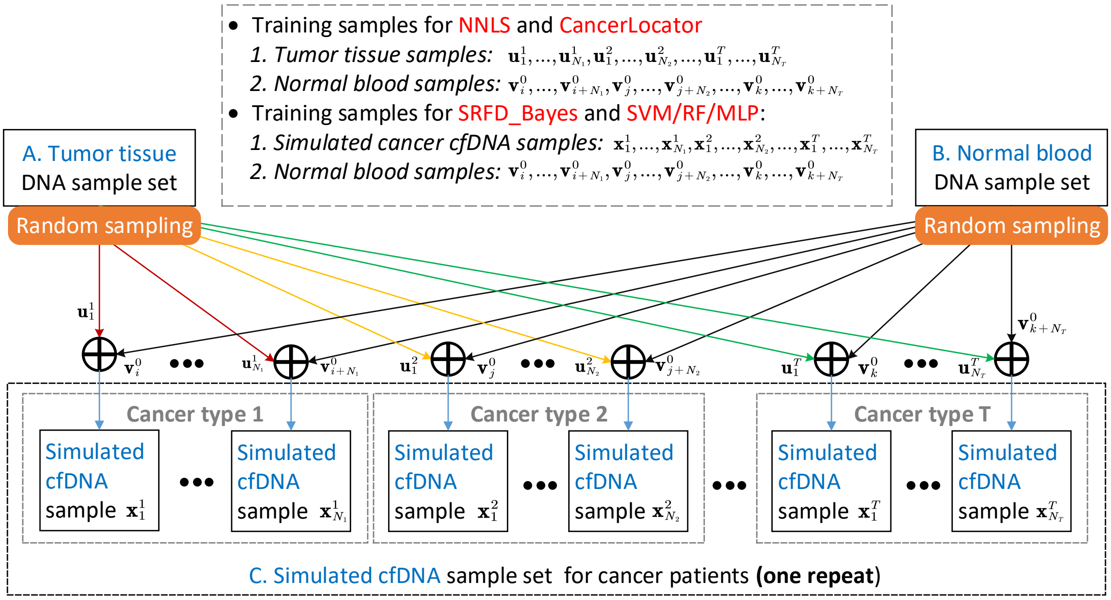

Supplementary Fig. 2 Visualization for the generation of simulated plasma data. Considering the copy number variant (CNV) events, the methylation level of simulated cfDNA sample on each methylation site can be given by Equation S1. Tumor tissue samples and normal blood samples are applied as training samples for the reference-based approaches, including NNLS and CancerLocator. The simulated cancer cfDNA samples and normal blood samples are exploited by SRFD-Bayes to directly learn a reference for Bayesian diagnostic model training, and simultaneously employed by other machine learning approaches, including support vector machine (SVM), random forest (RF), and multi-layer perception (MLP), to build corresponding diagnostic models.

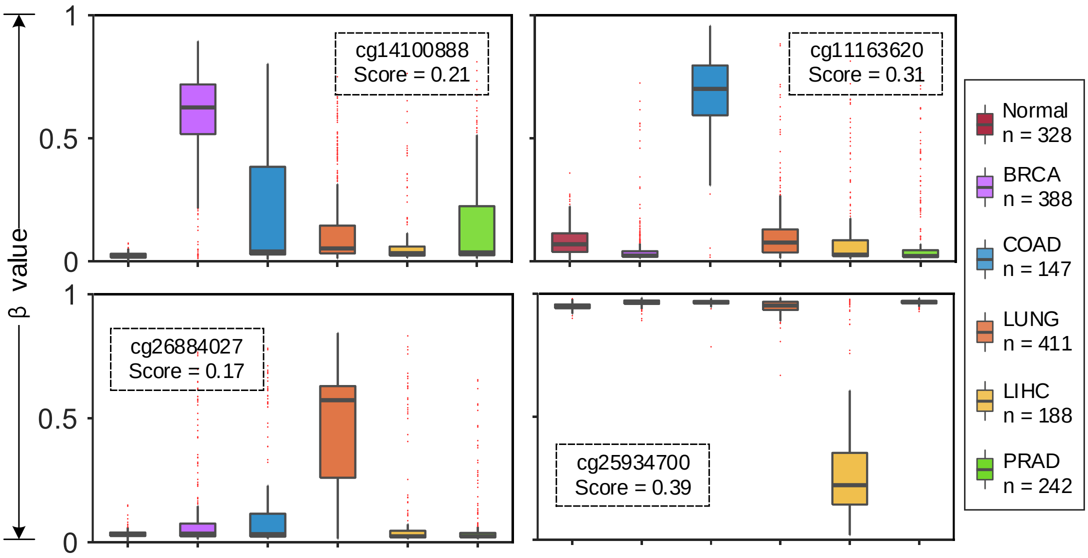

Supplementary Fig. 3 Top-1 TS methylation markers for different tumor type, including BRCA, COAD, LUNG and LIHC. The boxes are bounded by the first and third quartile with a horizontal line at the median and whiskers extend to the maximum and minimum values. Source data are provided as a Source Data file. n denotes the number of independent samples.

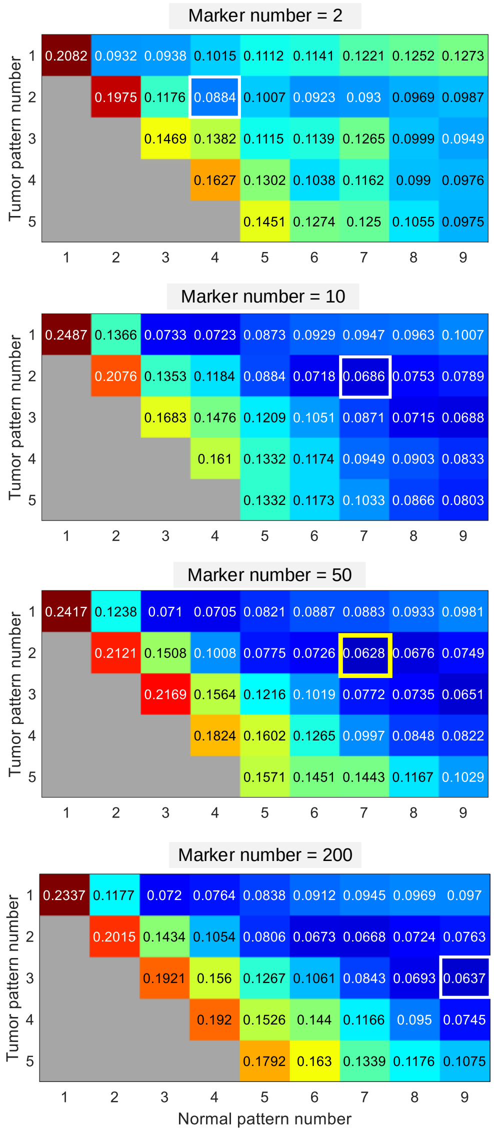

Supplementary Fig. 4 Experimental results on parameter configuration. The root mean square error (RMSE) of predicted source fractions on the validation dataset that summarized in different configurations of pattern number and marker number. White/yellow boxes suggest the minimum RMSE in each experimental group. The best performance is achieved when using Top-50 markers, 7 normal patterns and 2 tumor patterns.

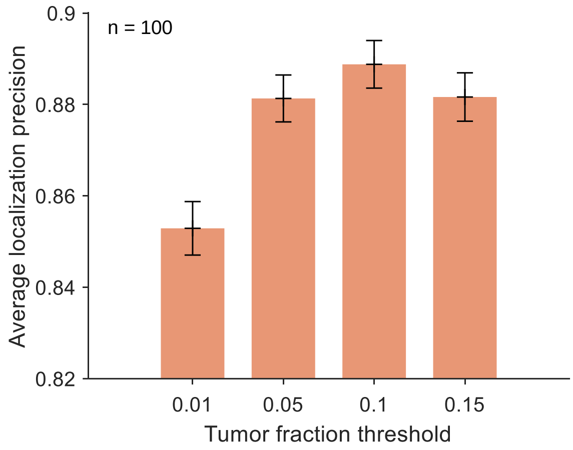

Supplementary Fig. 5 Experimental results on parameter study of CancerLocator. A parameter study of CancerLocator on a critical parameter, which acted as a tumor fraction threshold to distinguish cancer patients from normal controls, was implemented to achieve its best performance in simulation dataset. Error bars (in mean and standard deviation) were obtained by statistically repeating experiments 100 times. Source data are provided as a Source Data file.

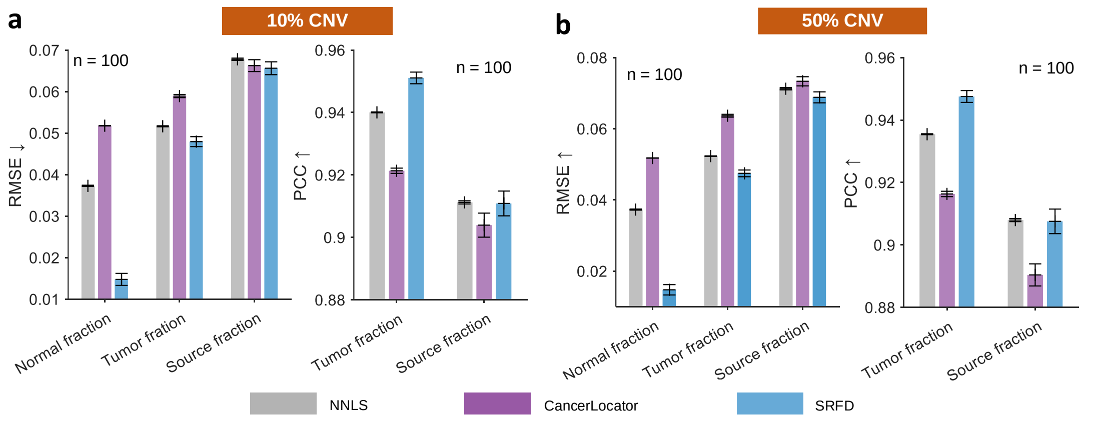

Supplementary Fig. 6 Comparison of overall deconvolution performance on simulation datasets with 10% CNV events and 50% CNV events. The performance was evaluated by the predicted normal fractions for healthy individuals, source fractions and tumor fractions for cancer patients, respectively. We repeated every experimental group 100 times, each with a random training dataset, to determine the average performance as well as the robustness between different approaches. Error bars (in mean and standard deviation) were obtained by statistically repeating experiments 100 times. Source data are provided as a Source Data file.

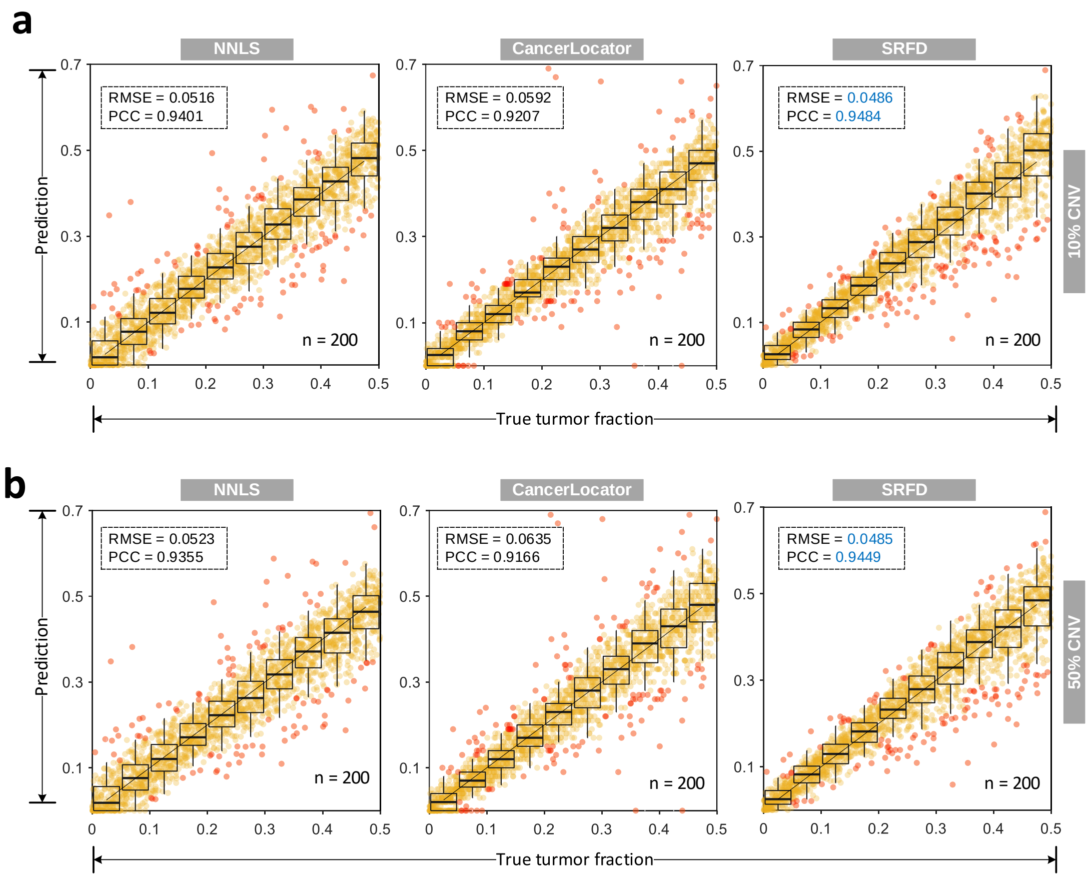

Supplementary Fig. 7 The comparison of detailed deconvolution performance achieved by different approaches on simulation tumor samples with 10% CNV events and 50% CNV events. Scatter and box plots exhibit the correlations between predicted tumor fractions and their ground truth, in which the black lines and red points denote $y = x$ and outliers, respectively. Blue font suggests the best performance. n = 200 independent experiments for each box. The boxes are bounded by the first and third quartile with a horizontal line at the median and whiskers extend to the maximum and minimum values. Source data are provided as a Source Data file.

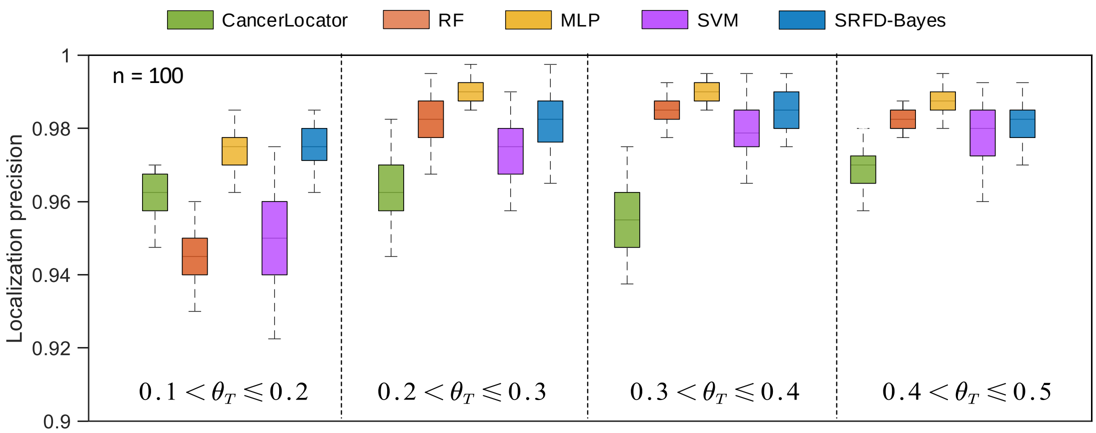

Supplementary Fig. 8 Comparison of localization performance on cancer samples with tumor fraction more than 0.1 ($\theta_T > 0.1$). The performance is evaluated in four intervals with (0.1, 0.2], (0.2, 0.3], (0.3, 0.4], (0.4, 0.5], and compared between our approach (SRFD-Bayes) as well as other approaches, including CancerLocator, RF, MLP and SVM. n = 100 independent experiments for each box. The boxes are bounded by the first and third quartile with a horizontal line at the median and whiskers extend to the maximum and minimum values. Source data are provided as a Source Data file.

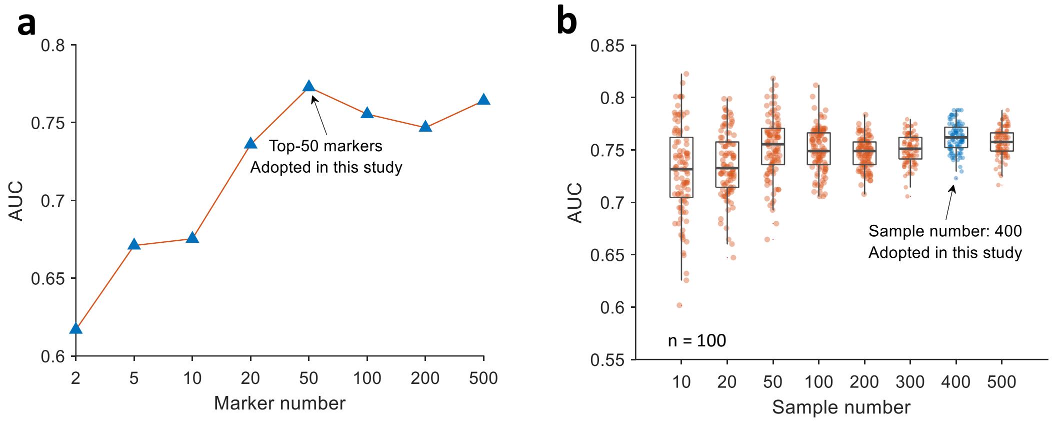

Supplementary Fig. 9 AUC performance comparison on patient cfDNA when using different number of samples and markers for training of SRFD. The performance evaluation of classifying cirrhosis patients and the patients with both cirrhosis as well as HCC when using different number of markers (**a**) and samples (**b**) for training of SRFD. **a** The AUC values calculated by exploiting predicted tumor fractions to classify cirrhosis patients and the patients with both cirrhosis and HCC increased as the number of markers grew, and then gradually leveled off when more than Top-50 markers were adopted. The best performance of 0.758 was achieved by employing the Top-50 markers, which was consistent with the experimental results on simulation dataset in Fig. 2d. **b** When fixing the marker number as Top-50, the mean AUC values and its robustness improved as the sample number grew. The best mean AUC of 0.758 was achieved at the number of 400 samples for each category, which also matched the diagnostic results shown in Fig. 2e. n = 100 independent experiments. The boxes are bounded by the first and third quartile with a horizontal line at the median and whiskers extend to the maximum and minimum values. Source data are provided as a Source Data file.

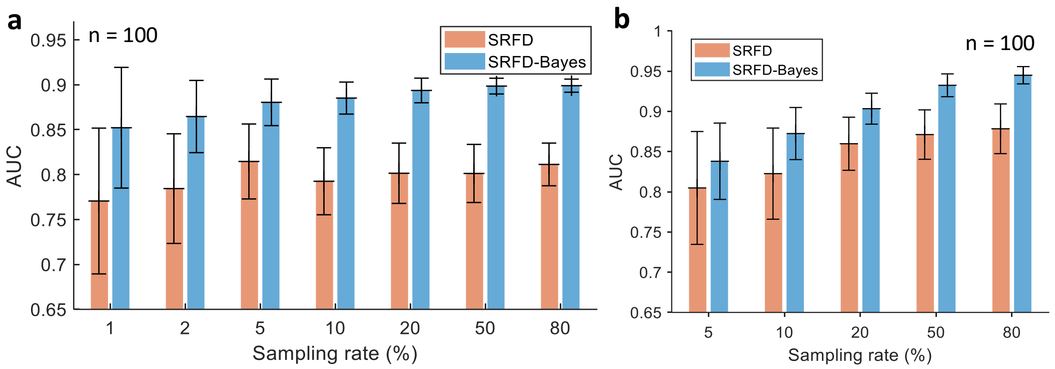

Supplementary Fig. 10 AUC performance comparison of distinguishing cancer patients from normal controls when using different number of samples for training of SRFD and SRFD-Bayes. a Experimental results for Xu et al. dataset (the number of entire training samples from cancer patients is 704). **b** Experimental results for Chen et al. dataset (the number of entire training samples from cancer patients is 113). The training cases were randomly resampled from the entire training dataset (from 1% to 80%), each with 100 repeats. The statistical performance indicated that the mean AUC values of both SRFD and SRFD-Bayes improved as the number of samples increased, while their standard deviation gradually reduced, suggesting a more robust performance. Error bars (in mean and standard deviation) were obtained by statistically repeating experiments 100 times. Source data are provided as a Source Data file.

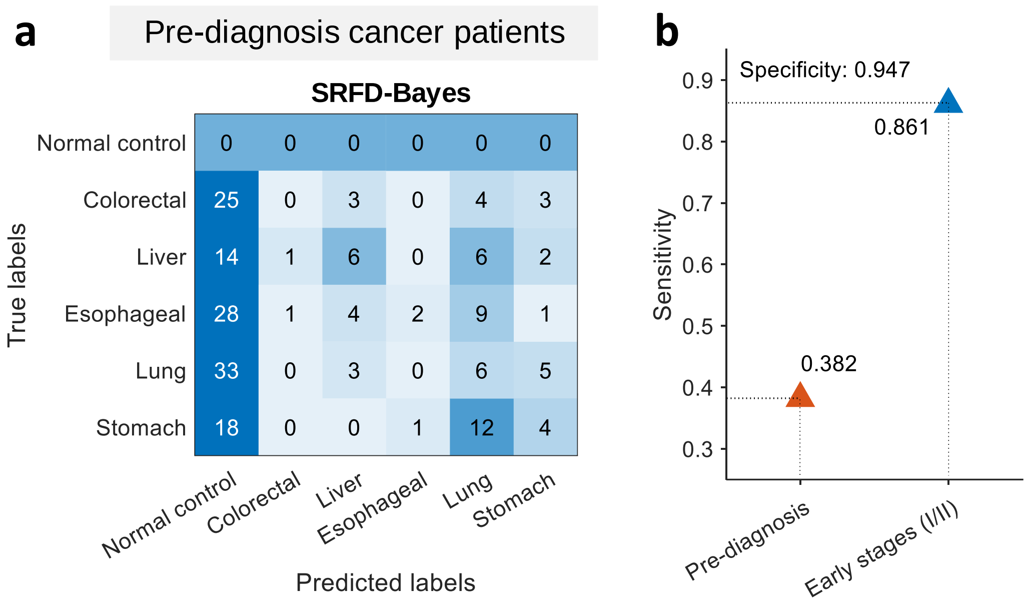

Supplementary Fig. 11 Diagnostic performance on pre-diagnosis patients. a Diagnostic result, visualized by confusion matrix, on pre-diagnosis patients. Only late-stage patients and a random half of normal controls are utilized as training samples for model establishment. **b** The detection sensitivity of pre-diagnosis participants and early-stage patients at a specificity of 94.7%

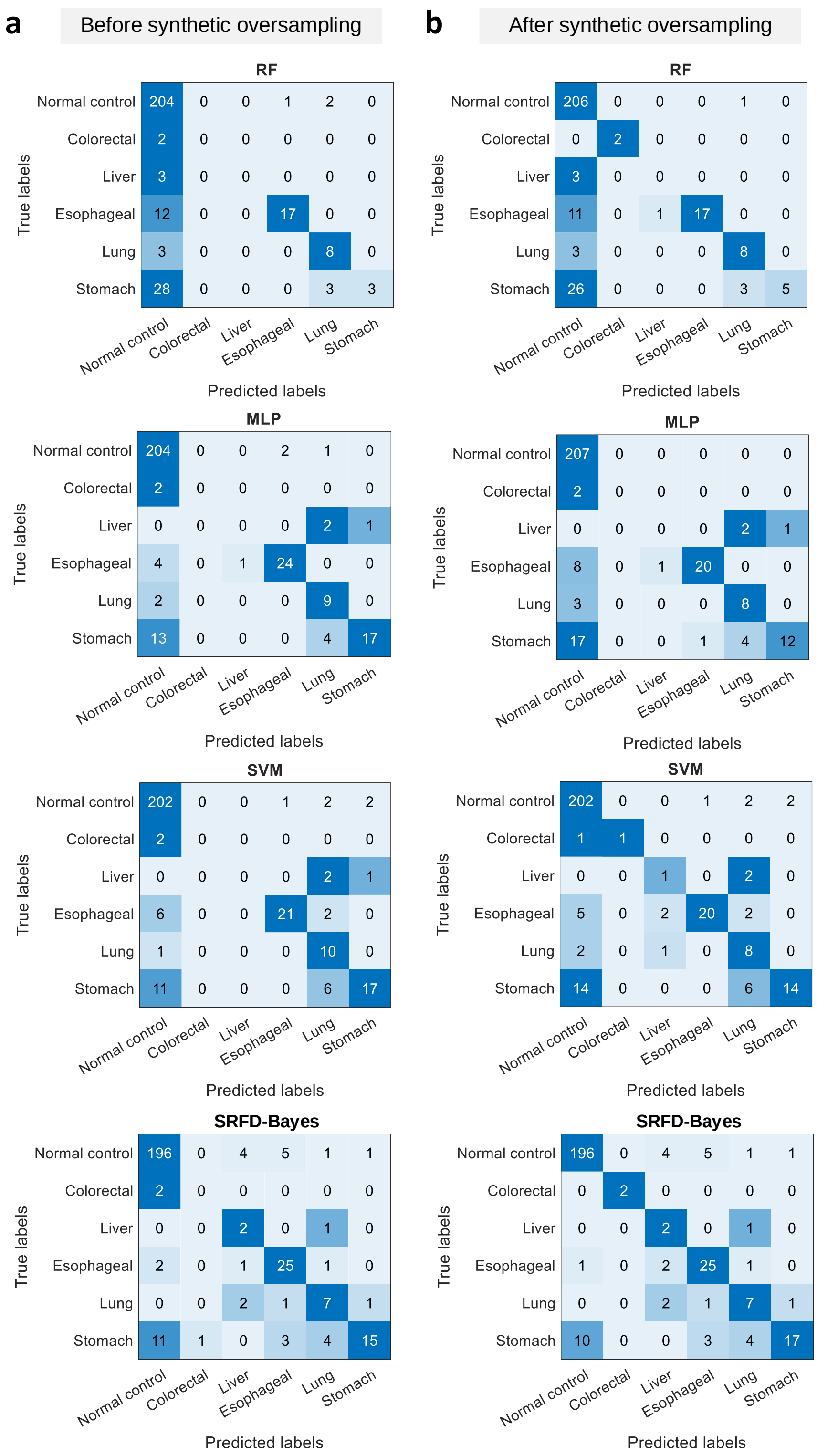

Supplementary Fig. 12 Diagnostic results before and after synthetic oversampling strategy. The diagnostic results are illustrated by confusion matrix before (**a**) and after (**b**) synthetic oversampling, on normal controls and early-stage patients. Only late-stage patients and a random half of normal controls are utilized as training samples for model establishment.

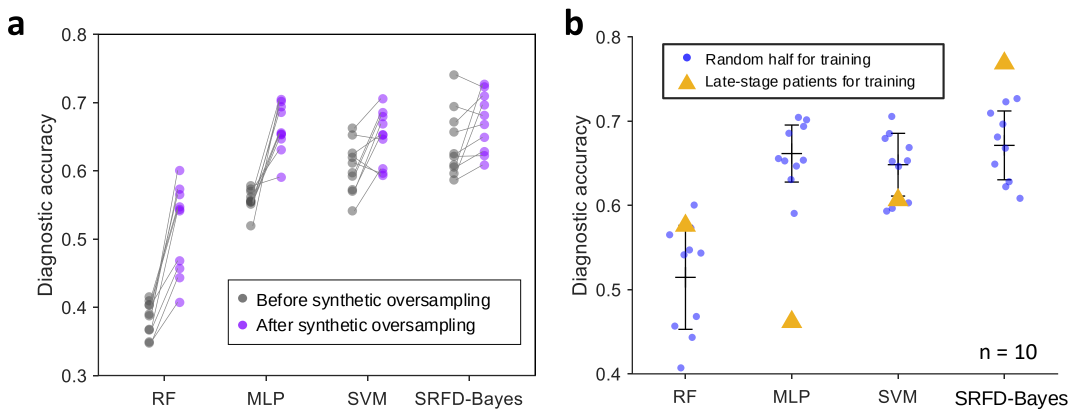

Supplementary Fig. 13 The comparison of diagnostic performance among different approaches. a The diagnostic accuracy of all approaches validated on test dataset before (gray) and after (purple) the synthetic oversampling strategy, in which the training dataset contains a random half of each category. Each line paired with two dots suggests a repeated experiment (n = 10). **b** The comparison of diagnostic accuracy between two different situations (after synthetic oversampling), where a random half (blue dots) or late-stage patients (yellow triangles) are utilized for training. Error bars (in mean and standard deviation) were obtained by statistically repeating experiments 10 times. Source data are provided as a Source Data file.

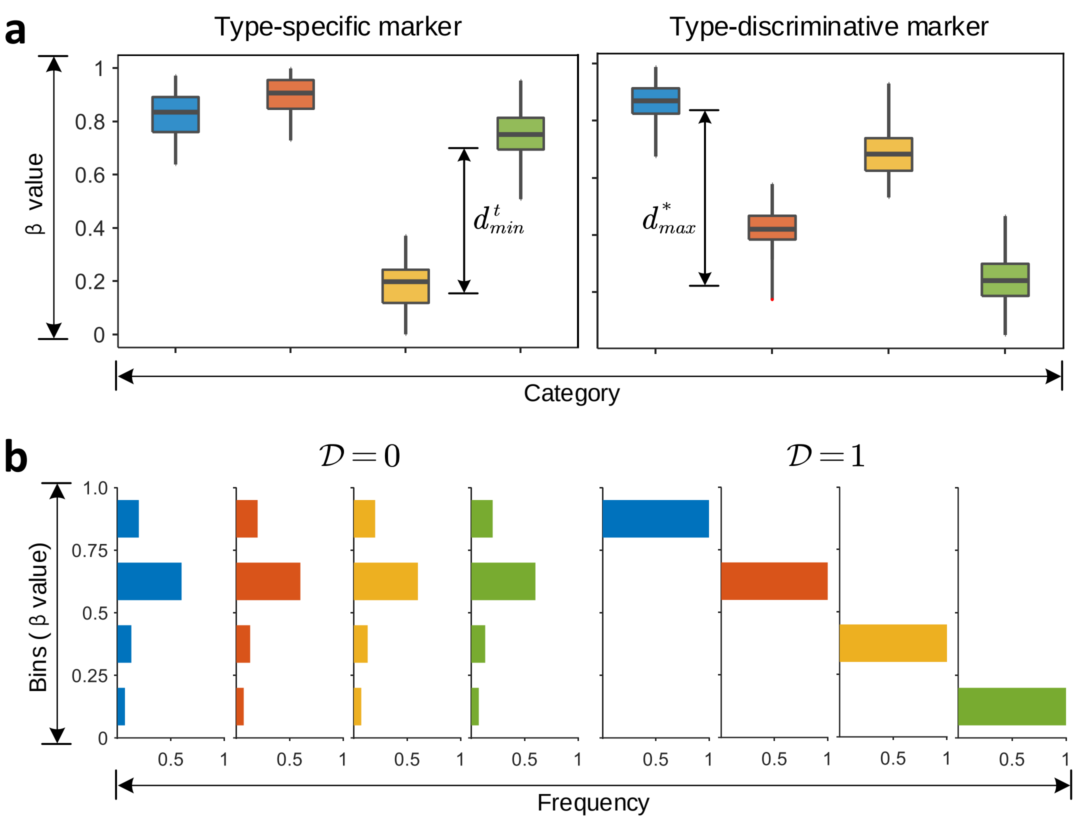

Supplementary Fig. 14 The schematic diagram of TS and TD markers as well as the visualization of the discriminability. a The schematic diagram of TS and TD markers. **b** The visualization of the two extreme situations where the discriminability $\mathcal{D} = 0$ or $\mathcal{D} = 1$. The boxes are bounded by the first and third quartile with a horizontal line at the median and whiskers extend to the maximum and minimum values.

References

1. Chabon, J. J. et al. Integrating genomic features for non-invasive early lung cancer detection. *Nature* **580**, 245–251 (2020).
2. Sun, K. et al. Plasma DNA tissue mapping by genome-wide methylation sequencing for noninvasive prenatal, cancer, and transplantation assessments. *Proc. Natl. Acad. Sci. USA* **112**, E5503–E5512 (2015).
3. Smyth, G. K. Linear models and empirical Bayes methods for assessing differential expression in microarray experiments. *Stat. Appl. Genet. Mol. Biol.* **3**, 1–25 (2004).
4. Xia, D. et al. Minimalist approaches to cancer tissue-of-origin classification by DNA methylation. *Mod. Pathol.* **33**, 1874–1888 (2020).
5. Newman, A. M. et al. Robust enumeration of cell subsets from tissue expression profiles. *Nat Methods* **12**, 453–457 (2015).
6. Baron, M. *et al.* A single-cell transcriptomic map of the human and mouse pancreas reveals inter-and intra-cell population structure[J]. *Cell Syst.* **3,** 346-360 (2016).
7. Hannum, G. et al. Genome-wide methylation profiles reveal quantitative views of human aging rates. *Mol. Cell* **49**, 359–367 (2013).
8. Moss, J. et al. Comprehensive human cell-type methylation atlas reveals origins of circulating cell-free DNA in health and disease. *Nat. Commun.* **9**, 5068 (2018).
9. Kang, S. et al. CancerLocator: non-invasive cancer diagnosis and tissue-of-origin prediction using methylation profiles of cell-free DNA. *Genome Biol.* **18**, 53 (2017).
10. Gordevičius, J. et al. Cell-free DNA modification dynamics in abiraterone acetate-treated prostate cancer patients. *Clin. Cancer Res.* **24**, 3317–3324 (2018).
11. Hlady, R. A. et al. Genome-wide discovery and validation of diagnostic DNA methylation-based biomarkers for hepatocellular cancer detection in circulating cell free DNA. *Theranostics* **9**, 7239–7250 (2019).
12. Chen, X. et al. Non-invasive early detection of cancer four years before conventional diagnosis using a blood test. *Nat. Commun.* **11**, 3475 (2020).
13. Xu, R. et al. Circulating tumour DNA methylation markers for diagnosis and prognosis of hepatocellular carcinoma. *Nat. Mater.* **16**, 1155–1161 (2017).
