# Supplementary material for: Tumor fractions deciphered from circulating cell-free DNA methylation for cancer early diagnosis
Source: Nat Commun. 2022 Dec 13;13:7694. doi: 10.1038/s41467-022-35320-3 (PMC9744803; doi:10.1038/s41467-022-35320-3)
Supplement: Supplementary file 2 — Reporting Summary [file 41467_2022_35320_MOESM2_ESM.pdf]

nature portfolio

Corresponding author(s): Min Liu, Junzhang Tian, Weibin Cheng

Last updated by author(s): Nov 17, 2022

Reporting Summary

Nature Portfolio wishes to improve the reproducibility of the work that we publish. This form provides structure for consistency and transparency in reporting. For further information on Nature Portfolio policies, see our Editorial Policies and the Editorial Policy Checklist.

Statistics

For all statistical analyses, confirm that the following items are present in the figure legend, table legend, main text, or Methods section.

n/a	Confirmed	
☐	☒	The exact sample size (n) for each experimental group/condition, given as a discrete number and unit of measurement
☐	☒	A statement on whether measurements were taken from distinct samples or whether the same sample was measured repeatedly
☐	☒	The statistical test(s) used AND whether they are one- or two-sided<br>*Only common tests should be described solely by name; describe more complex techniques in the Methods section.*
☐	☒	A description of all covariates tested
☐	☒	A description of any assumptions or corrections, such as tests of normality and adjustment for multiple comparisons
☐	☒	A full description of the statistical parameters including central tendency (e.g. means) or other basic estimates (e.g. regression coefficient) AND variation (e.g. standard deviation) or associated estimates of uncertainty (e.g. confidence intervals)
☐	☒	For null hypothesis testing, the test statistic (e.g. F, t, r) with confidence intervals, effect sizes, degrees of freedom and P value noted<br>*Give P values as exact values whenever suitable.*
☐	☒	For Bayesian analysis, information on the choice of priors and Markov chain Monte Carlo settings
☐	☒	For hierarchical and complex designs, identification of the appropriate level for tests and full reporting of outcomes
☐	☒	Estimates of effect sizes (e.g. Cohen's d, Pearson's r), indicating how they were calculated

Our web collection on statistics for biologists contains articles on many of the points above.

Software and code

Policy information about availability of computer code

Data collection	No software was used
Data analysis	We used MATLAB R2018b with Parallel Computing Toolbox 6.13, Statistics and Machine Learning Toolbox 11.4 and Deep Learning Toolbox 12.0. We used borderline-SMOTE algorithm to do oversampling. We used R packages (ChAMP) to preprocess the data from TCGA. SRFD-Bayes(https://github.com/Astaxanthin/SRFD-Bayes)

For manuscripts utilizing custom algorithms or software that are central to the research but not yet described in published literature, software must be made available to editors and reviewers. We strongly encourage code deposition in a community repository (e.g. GitHub). See the Nature Portfolio guidelines for submitting code & software for further information.

Data

Policy information about availability of data

All manuscripts must include a data availability statement. This statement should provide the following information, where applicable:

- Accession codes, unique identifiers, or web links for publicly available datasets
- A description of any restrictions on data availability
- For clinical datasets or third party data, please ensure that the statement adheres to our policy

The main data supporting the results are available within this article as well as its Supplementary information and Supplementary data. The cfDNA-SNV data analyzed in this study were collected from Lung-CLiP on Website https://clip.stanford.edu/. The DNA methylation profiles of tumor tissues adopted in this study

were collected from TCGA. The normal blood cfDNA methylation profiles were collected from GSE40279 (https://www.ncbi.nlm.nih.gov/geo/). The plasma cfDNA methylation profiles were collected from GSE122126, GSE108462, GSE129374 and the repository NCOMMS-20-10056-T on GitHub (https://github.com/ncomms-20-10056-t/ncomms-20-10056-t). The plasma samples with 10 methylation sites for HCC patients and normal controls are collected from the Supplementary materials of previous study.

Human research participants

Policy information about studies involving human research participants and Sex and Gender in Research.

Reporting on sex and gender	We used only publicly accessible data. Sex and gender were not considered in this study.
Population characteristics	We used only publicly accessible data. The tumor types and diagnosis stages of all cancer patients were used in this study.
Recruitment	We did not recruit any human research participants.
Ethics oversight	We used only publicly accessible data, which had already received ethical approval.

Note that full information on the approval of the study protocol must also be provided in the manuscript.

Field-specific reporting

Please select the one below that is the best fit for your research. If you are not sure, read the appropriate sections before making your selection.

☒ Life sciences ☐ Behavioural & social sciences ☐ Ecological, evolutionary & environmental sciences

For a reference copy of the document with all sections, see nature.com/documents/nr-reporting-summary-flat.pdf

Life sciences study design

All studies must disclose on these points even when the disclosure is negative.

Sample size	We used only publicly accessible data, including 656 normal blood samples from GSE40279, 12 normal cfDNA samples from GSE122126, 29 prostate cancer samples from GSE108462, 22 HCC&cirrhosis and 21 cirrhosis samples from GSE129374, 835 normal and 1050 HCC samples from Xu et al. dataset, 414 normal and 223 cancer samples from Chen et al. dataset. All the data come from publicly accessible datasets and sufficient for our study. The tissue data from TCGA and the normal blood data from GSE40279 and GSE122126 are sufficient to generate simulation datasets for the verification of our approach. The real plasma cfDNA datasets from GSE108462 and GSE129374 are sufficient to validate the reference obtained by our approach on the deconvolution of real plasma data. The real datasets from Xu et al. dataset and Chen et al. dataset are large enough to testify the performance of our approach on predicting tumor fractions and cancer early diagnosis.
Data exclusions	We did not use cancer samples from Moss, J. et al. dataset(GSE122126) since it lacked quantification results.
Replication	We directly applied the learned reference database (from TCGA, GSE40279 and GSE122126) to deconvolve the real plasma cfDNA methylation profiles (from GSE108462 and GSE129374) for replication. Deconvolution of cfDNA methylation from cancer patients in two independent real datasets (Xu et al. and Chen et al. datasets) was performed for replication. We repeat all the experiments in simulation datasets 100 times, each with a random dataset generation, to evaluate the average performance and the robustness of our approach.
Randomization	The simulation datasets were randomly split and repeated 100 times.
Blinding	We used only publicly accessible datasets for retrospective studies, which already have labels on each sample. No blinding was used. We random split each dataset into training and test subset and repeated 100 times to ensure a fair evaluation.

Reporting for specific materials, systems and methods

We require information from authors about some types of materials, experimental systems and methods used in many studies. Here, indicate whether each material, system or method listed is relevant to your study. If you are not sure if a list item applies to your research, read the appropriate section before selecting a response.

Materials & experimental systems

n/a	Involved in the study
☒	☐ Antibodies
☒	☐ Eukaryotic cell lines
☒	☐ Palaeontology and archaeology
☒	☐ Animals and other organisms
☒	☐ Clinical data
☒	☐ Dual use research of concern

Methods

n/a	Involved in the study
☒	☐ ChIP-seq
☒	☐ Flow cytometry
☒	☐ MRI-based neuroimaging
